# *OsAAI1* Increases Rice Yield and Drought Tolerance Dependent on ABA-Mediated Regulatory and ROS Scavenging Pathway

**DOI:** 10.1186/s12284-023-00650-3

**Published:** 2023-08-03

**Authors:** Qing Long, Shichun Qiu, Jianmin Man, Denghong Ren, Ning Xu, Rui Luo

**Affiliations:** 1 Key laboratory of Plant Resource Conservation and Germplasm Innovation in Mountainous Region (Ministry of Education), College of Life Sciences, Institute of Agro-bioengineering, Guizhou University, Guiyang, 550025 Guizhou Province China; 2 Chongqing Three Gorges Academy of Agricultural Sciences, Wanzhou, Chongqing City, 404155 China

**Keywords:** *OsAAI1*, Yield, Drought stress, Abscisic acid, ROS scavenging ability

## Abstract

In this study, we investigated the function of *OsAAI1* in yield and drought tolerance by constructing overexpression line OE-OsAAI1 and mutant line *osaai1*. Bioinformatics analysis showed that the AAI gene-*OsAAI1-* belongs to the HPS_like subfamily of the AAI_LTSS superfamily, and OsAAI1 was localized in the nucleus. The expression of *OsAAI1* was significantly induced by ABA and drought stress. *OsAAI1* overexpression (OE19) significantly increased, and gene mutant (*osaai1-1*) repressed plant height, primary root length, lateral root number, grain size and yield in rice. Moreover, physiological and biochemical analyses showed that *osaai1* was sensitive to drought stress, while OE19 enhanced the drought tolerance in rice. DAB and NBT staining revealed that under drought treatment, *osaai1* accumulated a large amount of ROS compared with the wild type, while OE19 accumulated the least, and CAT, APX, GPX, GR activities were higher in OE19 and lower in *osaai1*, suggesting that OE19 improves rice tolerance to drought stress by enhancing ROS scavenging ability. OE19 also induce the expression of ABA-mediated regulatory pathway genes and enhance accumulation of ABA content in rice seedling. Predictably, OE19 displayed enhanced sensitivity to ABA, and ROS accumulation was significantly higher than in wild type and *osaai1* under 3 µM ABA treatment. Thus, these results suggest that *OsAAI1* is a positive regulator of rice yield and drought tolerance dependent on the ABA-mediated regulatory and ROS scavenging pathway.

**Supplementary Information:**

The online version contains supplementary material available at 10.1186/s12284-023-00650-3.

## Background

Rice is one of the most important food crops in the world, and more than half of the world's population depends on rice as a staple food (Gross and Zhao 2014; Lyman et al. 2013). Rice requires large amounts of water to maintain normal growth and development (Lafitte et al. 2006). During the growth of rice, water deficiency can affect its yield and quality, and in severe cases can even lead to plant death (Krasensky and Jonak 2012). Drought is increasingly becoming one of the major causes of crop productivity loss due to global climate changes that have led to uneven distribution of water globally (Fukao and Xiong 2013). Therefore, it is a very urgent task to use the resilience of rice to ensure yield stability under unfavorable environmental conditions (Chaves et al. 2003).

Abscisic acid (ABA), a class of sesquiterpene carboxylic acids produced by oxidative cleavage of carotenoids, is a key hormone in plant growth and development (Li and Walton 1990). ABA not only plays a crucial role in seed dormancy, seed germination, root structure development, embryo maturation, and stomatal opening and closing (Dejonghe et al. 2018; Hsu et al. 2021), but also plays a prime mediator of drought stress (Boominathan et al. 2004). Numerous studies have shown that drought conditions induce the expression of ABA biosynthetic genes to accumulate ABA and regulate stomatal closure for the purpose of water conservation (Hsu et al. 2021; Iuchi et al. 2001; Murata et al. 2015).

Reactive oxygen species (ROS), which include the superoxide anion radical (O_2_^−^), hydroxyl radical (OH^−^), and hydrogen peroxide (H_2_O_2_), regulate plant growth and development, and protect against abiotic and biotic stresses (Mittler et al. 2011; Tanaka et al. 2006; Torres and Dangl 2005). Production of ROS is enhanced in plants after suffering from various abiotic stresses (such as drought, salt, and temperature) (Mittler et al. 2004). However, excessive accumulation of ROS is harmful to cells and causes oxidative damage to lipids, DNA, and proteins (Apel and Hirt 2004). To balance ROS production and destruction, plants have evolved an antioxidant system such as antioxidant enzymes system (Xu et al. 2013). ROS scavenging enzyme systems in plants mainly include catalase (CAT), ascorbate peroxidase (APX), glutathione peroxidase (GPX), glutathione reductase (GR) and glutathione sulfotransferase (GST) (Foyer and Noctor 2005; Mittler 2002; Xu et al. 2018). It has been suggested that there may be some mechanism by which ROS and ABA interact and regulate each other (Chen et al. 2022a, b). Overexpression of rice ABA receptor 6 (*OsPYL6*) can improve drought tolerance by increasing ABA content and positively regulating ROS detoxification and membrane stability (Santosh et al. 2021). Overexpression of UDP-glycosyltransferase (*UGT3*) can enhance drought tolerance through modulating ABA synthesis and scavenging ROS in rice (Wang et al. 2021). In addition, ROS are shown to participate in ABA-mediated stomatal closure (Postiglione and Muday 2020). The production and accumulation of apoplastic ROS depend on ABA signaling (Wu et al. 2020). ROS can enhance ABA signaling by acting as a second messenger, ROS coordinates with ABA to regulate stomatal closure in response to drought (Liu et al. 2022), suggesting both ABA and ROS are important in resisting stresses.

Alpha-Amylase Inhibitors (AAI), Lipid Transfer (LT) and Seed Storage (SS) Protein family (AAI_LTSS Protein family) is a family of proteins unique to higher plants, total contains 5 members, which named Alpha-Amylase Inhibitors (AAIs) and Seed Storage (SS) Protein subfamily (AAI_SS), Hydrophobic Protein from Soybean (HPS)-like subfamily, Non-specific lipid-transfer protein type 2 (nsLTP2) subfamily, Non-specific lipid-transfer protein type 1 (nsLTP1) subfamily and Non-specific lipid-transfer protein (nsLTP)-like subfamily respectively. Proteins in this family not only play important roles in defending plants from insects and pathogens, but also involved in lipid transport between intracellular membranes, and nutrient storage (Kader 1996; Kreis et al. 1985; Wirtz 1991). *AAI* genes-belongs to the AAI_LTSS superfamily-encode three domains including LTP2 domain, hydrophobic seed domain, and trypsin alpha amylase domain (Lu et al. 2020; Qanmber et al. 2019). The LTP2 domain is characterized by containing an eight cysteine pattern (8CM) backbone as shown below, C-Xn-C-Xn-CC-Xn-CXC-Xn-C-Xn-C (Fleury et al. 2019; Jose-Estanyol et al. 2004). The positions of the eight cysteine residues follow a conservative pattern, in which the third and fourth cysteines are adjacent in the polypeptide chain, and the fifth and sixth cysteines are divided by only one residue (Jose-Estanyol et al. 2004). Plant sequences possessing this motif belong to various proteins with different functions, including enzyme inhibition, lipid transfer, cell wall structure and storage protection (Barciszewski et al. 2000; Henrissat et al. 1988; Jose-Estanyol et al. 2004; Shewry et al. 1995; Shewry and Tatham 1990). However, there were few functions reported in *AAI* genes with the hydrophobic seed domain or trypsin alpha amylase domain for now. In a recent study, cotton *GhAAI66* protein was found to contain LTP2 domain, hydrophobic seed domain and trypsin alpha amylase domain and induced early flowering in cotton (Qanmber et al. 2019). The HPS_like subfamily, composed of proteins with similarity to Hydrophobic Protein from Soybean (HPS), is a small hydrophobic protein with unknown function related to cereal-type alpha-amylase inhibitors and lipid transfer proteins (Baud et al. 1993). Except for HPS, members of this subfamily include a dark-inducible protein (*LeDI-2*) from *Lithospermum erythrorhizon* (Yazaki et al. 2001), a hybrid proline-rich protein (*HyPRP*) from maize (Jose-Estanyol et al. 1992), rice *RcC3* protein (Xu et al. 1995), and maize *ZRP3* protein (John et al. 1992). *HyPRP* is an embryo-specific protein that contains an N-terminal proline-rich domain and a C-terminal HPS-like cysteine-rich domain (Jose-Estanyol et al. 1992). It has been suggested that *HyPRP* may be involved in the stability and defense of the developing embryo (Jose-Estanyol et al. 1992). *LeDI-2* is a root-specific protein that may be involved in regulating the biosynthesis of shikonin derivatives in *L. erythrorhizon* (Yazaki et al. 2001). Maize *ZRP3* and rice *RcC3* are root-specific proteins (John et al. 1992; Xu et al. 1995). Overexpression of *RCc3* gene accelerates root growth hormone transport and increases the rate of growth hormone biosynthesis in rice root, which improves the root structure and enhances plant tolerance to salt stress (Xu et al. 1995).

In this study, we determined and functionally characterized an AAI gene that belongs to the HPS_like subfamily, named it as *OsAAI1* (LOC_Os04g55159) in rice. Our results provide a theoretical basis for developing drought-resistant and high-yield transgenic rice with a potential application value.

## Results

### Bioinformatics Analysis of *OsAAI1*

The amino acid sequence of the rice *OsAAI1* gene was retrieved and downloaded from the NCBI website. After NCBI blast online comparison, 22 and 9 homologous genes were screened in *Oryza sativa* L. and *Arabidopsis*, the physiological and biochemical properties of these homologs were analyzed and speculated by the online protein physicochemical property prediction website ExPasy ProtParam (Additional file 1: Table S1). The conserved structural domains of the OsAAI1 proteins were also analyzed using the online software SMART (http://smart.embl-heidelberg.de/), and the results showed that the conserved AAI domain were contained at amino acids 211–289. Further validation of the conserved structural domain at NCBI CDsearch revealed that the protein belongs to the HPS_like subfamily of the AAI_LTSS superfamily (Additional file 2: Fig. S1).

**Fig. 1:**
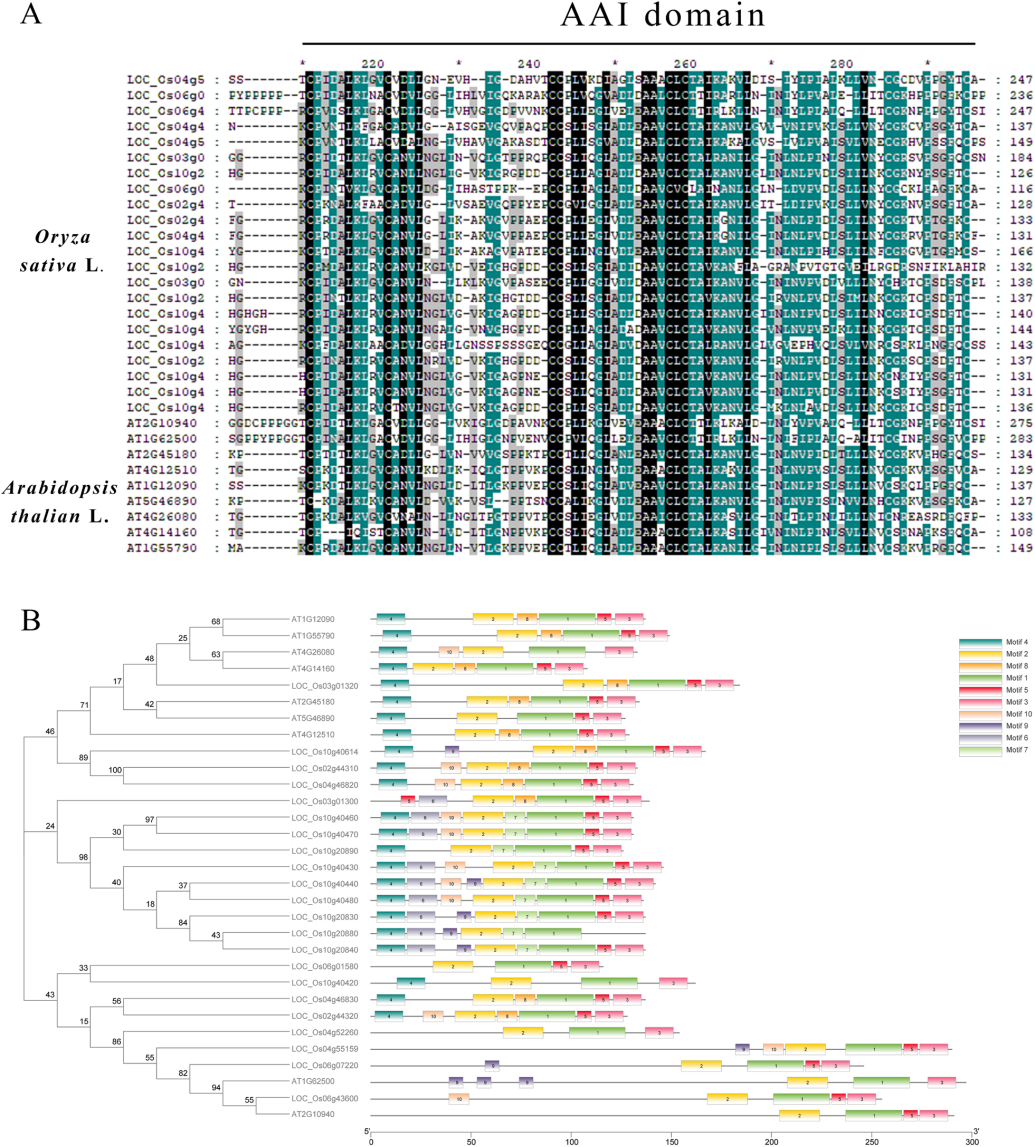
Phylogenetic tree and homologous sequence alignment of OsAAI1. **A** Homology comparison of OsAAI1 with Amino Acid Sequences in *Oryza sativa* L. and *Arabidopsis* (All sequence). Black means the conservative rate is 100%, green represents a conservative rate of 70–100% (excluding 100%), grey represents a conservative rate of 50–70% (excluding 70%). **B** Evolutionary tree of *OsAAI1* homologous genes in *Oryza sativa* L. and *Arabidopsis*.

Sequence alignment of the amino acid sequence of OsAAI1 with other members of the AAIs family in *Oryza sativa* L. and *Arabidopsis thaliana* using the software GeneDOC showed that OsAAI1 shares a highly conserved AAI domain with all these proteins (Fig. 1A). The OsAAI1 protein sequences retrieved on NCBI were analyzed by BLAST alignment with some of the AAIs family proteins from the Pteridophyta, the monocotyledonous plant *Oryza sativa* and *Zea mays*, and the dicotyledonous plant *Glycine max* and *Arabidopsis thaliana*, and 100 homologous proteins were screened. The online software MEME was used to analyze the 100 homologous proteins, the common structural sequences of all proteins in motif 1,2,3 were found among all motifs (Fig. 1B, Additional file 2: Fig. S2A–C). MEGA 7.0 software was used to construct an evolutionary tree for the 100 homologous proteins and OsAAI1 proteins. Then the constructed evolutionary tree and the protein conserved structural domain elements were combined and touched up by TBtools software to obtain the phylogenetic tree with protein conserved structural domain elements (Fig. 1B, Additional file 2: Fig. S2D).

### Tissue-Specific and Stress-Responsive Expression of *OsAAI1*

**Fig. 2:**
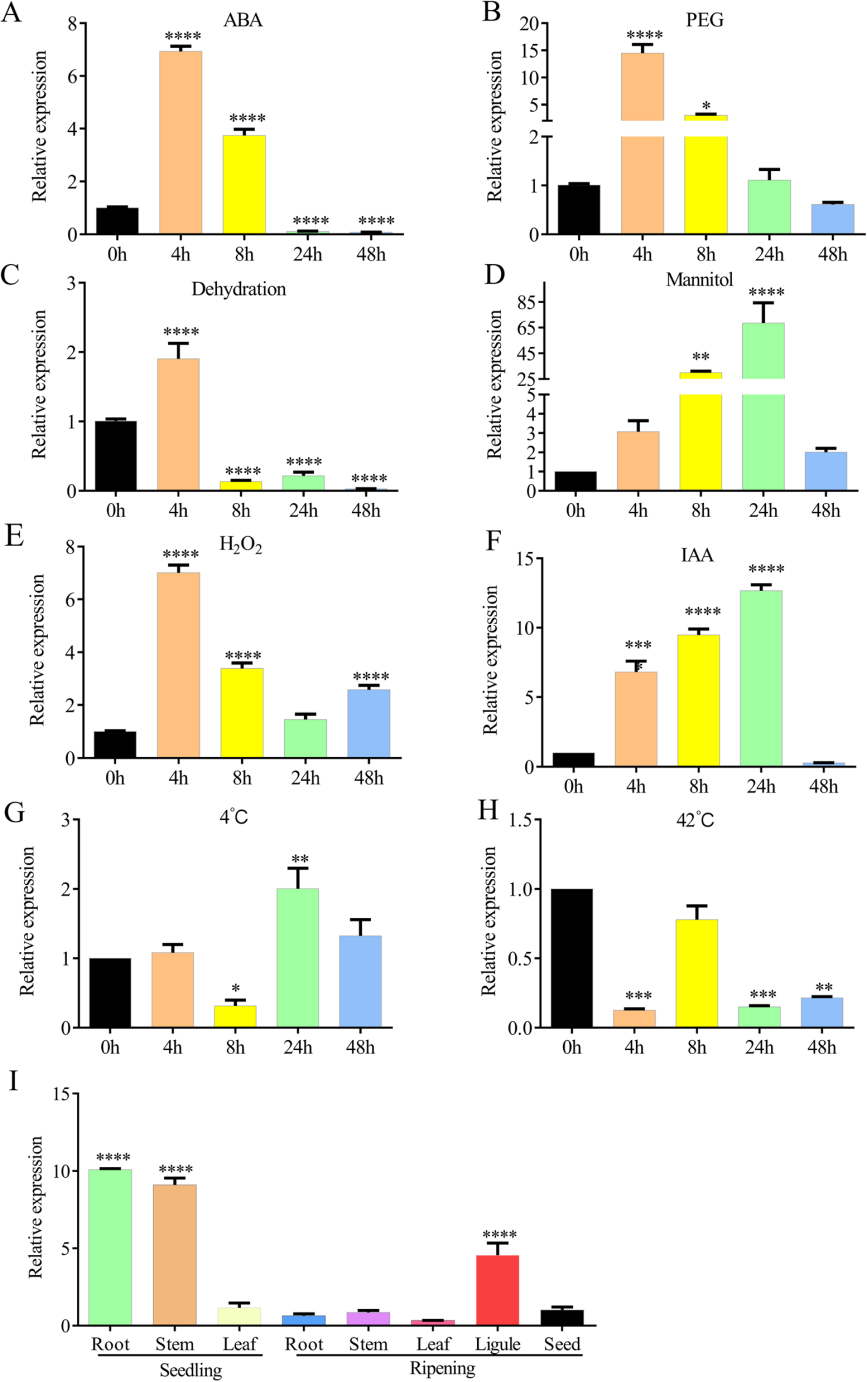
Response of *OsAAI1* to stress and hormones and its differential expression in space and time. **A–H** Changes in *OsAAI1* expression from 0 h to 48 h after 100 mM Mannitol, 4 °C, 42 °C, 20% PEG, dehydration, 1 mM H_2_O_2_, 10 nM IAA, and 100 µM ABA treatments. **I** Spatiotemporal differential expression of *OsAAI1* in rice. (Asterisks indicate a statistically significant difference compared with ZH11. **P < 0.01, ***P < 0.001, ****P < 0.0001; One-way ANOVA)

The promoter analysis of *OsAAI1* found that the promoter contained abscisic acid (ABA) response elements, MYB binding site involved in drought-inducibility and MYBHv1 binding sit, suggesting that *OsAAI1* may be induced by ABA and drought stress during rice growth and development (Additional file 2: Fig. S3E). To further confirm the effects of phytohormones and abiotic stresses on *OsAAI1* expression, qRT-PCR was applied to examine relative expression levels of *OsAAI1*. The results showed that *OsAAI1* can be induced by ABA, IAA, dehydration, PEG, H_2_O_2_, mannitol, low temperature (4 °C) and high temperature (42 °C) in rice at different time points after treatment, implying that *OsAAI1* is responsive to abiotic stress and ABA pathway (Fig. 2A–H). To determine the spatiotemporal expression pattern of *OsAAI1* under normal growth conditions. We extracted total RNA from different periods and different parts of rice and performed qRT-PCR analysis. The data indicated that *OsAAI1* was expressed in all of tissues tested and showed higher levels in young root compared with other tissues (Fig. 2I), suggesting that *OsAAI1* gene may play an important role in rice root development. This is basically the same as our predictive analysis results (Additional file 2: Fig. S3A–D).

### Subcellular Localization of OsAAI1

**Fig. 3:**
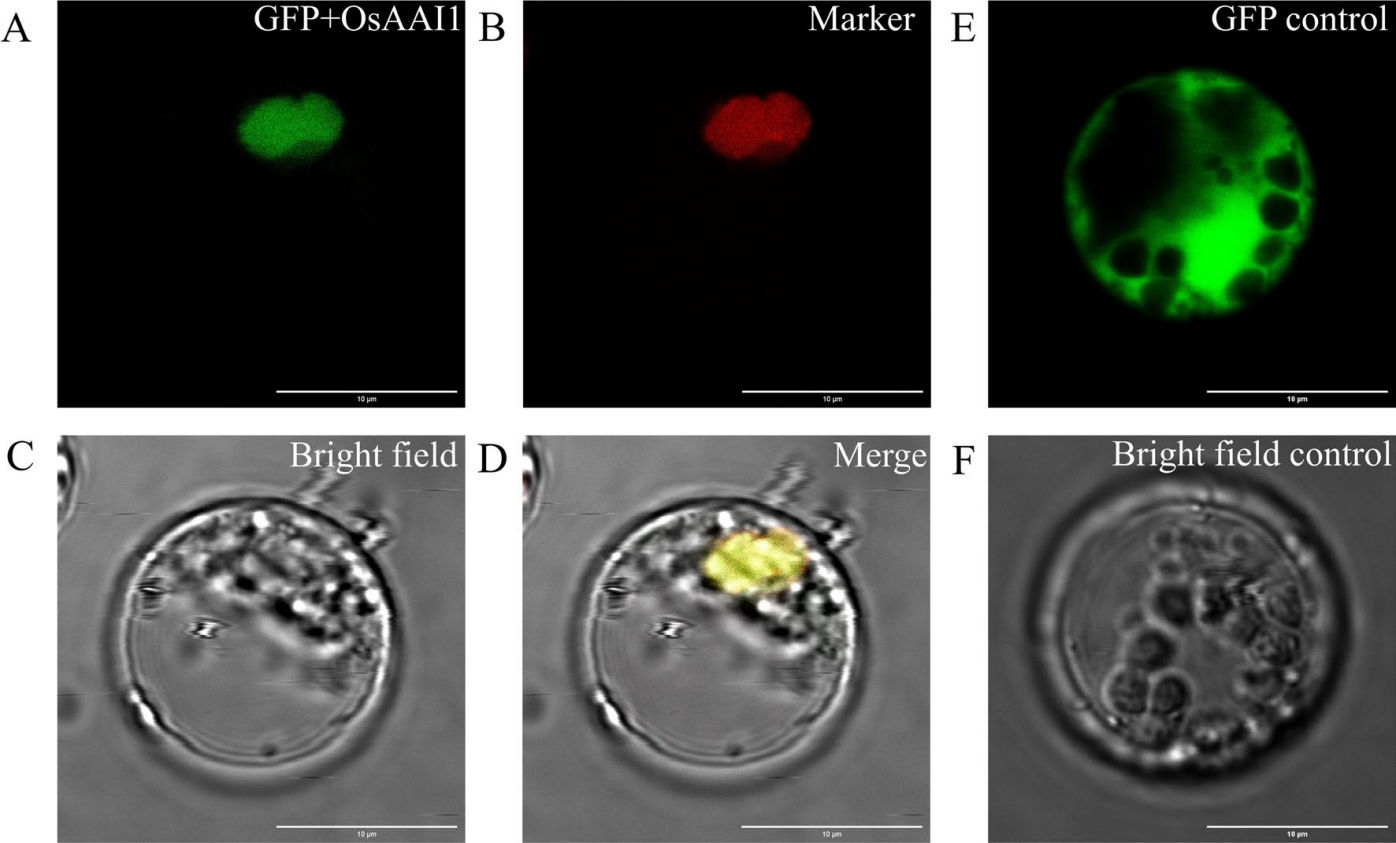
OsAAI1 subcellular localization. Empty vector 35S::GFP and the 35S::OsAAI1-GFP fusion were transiently expressed in rice protoplasts. The right panels show the fluorescent signal from the unfused GFP control (**E**, **F**), which is distributed throughout the cell. The left panels show the fluorescent signal from OsAAI1-GFP **A–D**, which localizes in the nucleus. (GFP fluorescence was detected at 488 nm excitation and 561 nm emission wavelength. Bars = 10 µm)

In order to detect the subcellular localization of OsAAI1 in plant cells, CDS sequence of *OsAAI1* was fused with a Green Fluorescent Protein (GFP) reporter gene and transiently expressed in rice protoplast under the control of a strong 35S promoter. The results showed that unlike the signal for free GFP, which was found in the whole cell, the fluorescence signal of the OsAAI1-GFP fusion protein was localized in the nucleus (Fig. 3). This is basically the same as our predictive analysis results (Additional file 1: Table S1). This result indicates that *OsAAI1* may have multiple functions in rice.

### Phenotypic Analysis of Transgenic Lines at Seedling Stage

**Fig. 4:**
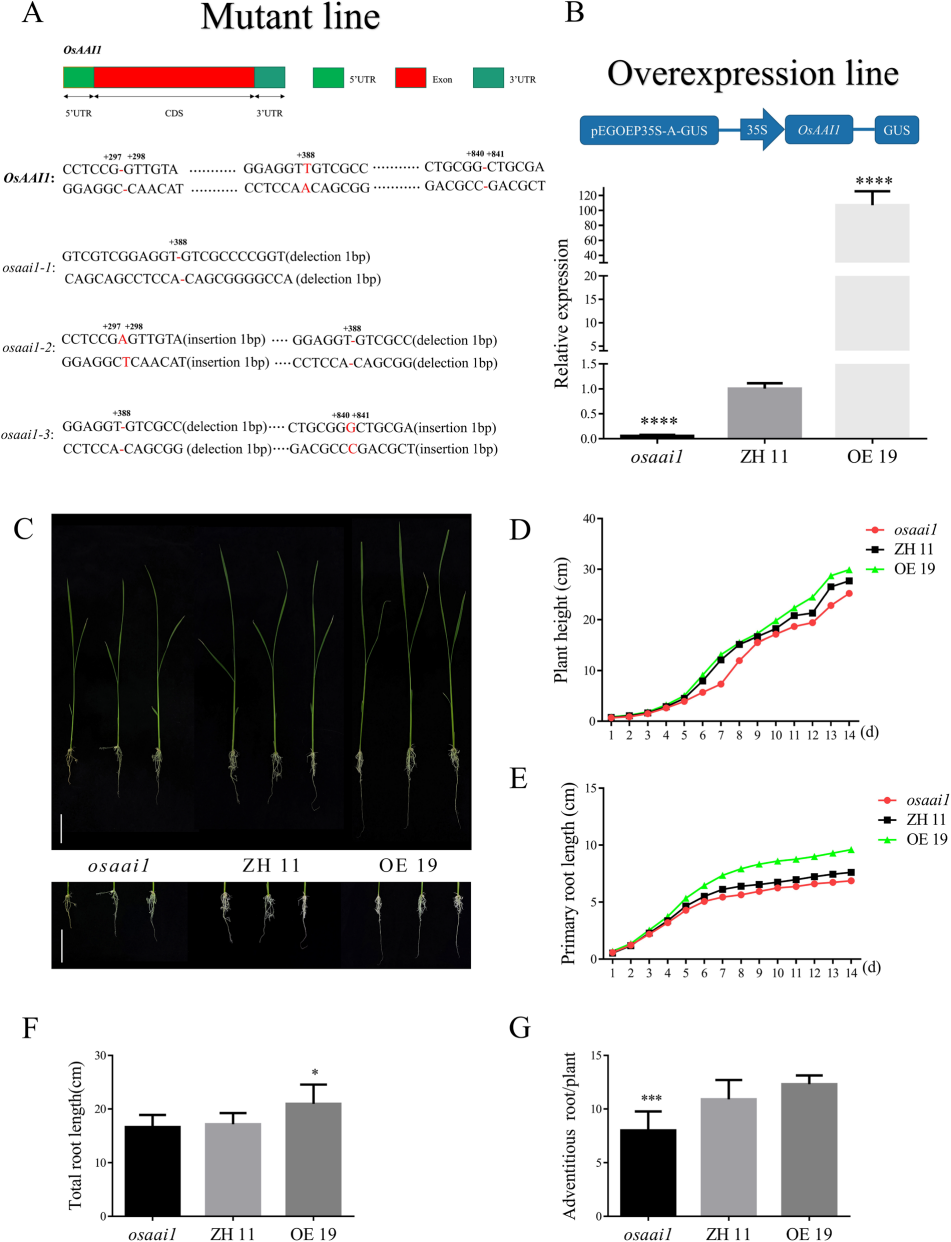
Phenotypic analysis of transgenic lines at seedling stage. **A** Schematic diagram of *OsAAI1* gene structure of mutant lines (*osaai1-1*, *osaai1-2* and *osaai1-3*). **B** Schematic diagram of *OsAAI1* gene structure of overexpression lines and relative expression of *osaai1*, ZH11, and OE19 using qRT-PCR analysis. **C** Phenotypes of *osaai1*, ZH11, and OE19 grown normally for 14 days. Bars = 4.5 cm **D** Plant height (cm), **E** Primary root length (cm), **F** Total root length (cm) and **G** Adventitious root/plant(n) of *osaai1*, ZH11, and OE19 tested in C. (Asterisks indicate a statistically significant difference compared with ZH11. **P < 0.01, ***P < 0.001, ****P < 0.0001; One-way ANOVA).

To further explore the function of *OsAAI1* in rice growth and development, we constructed *OsAAI1* gene editing vector and overexpression vector through molecular biology, and obtained *osaai1* mutant lines (*osaai1-1*, *osaai1-2*, *osaai1-3*) and overexpression lines (OE2, OE3, OE6, OE9, OE18, OE19) through genetic transformation. Sequence analysis showed that *osaai1-1*, *osaai1-2*, *osaai1-3* all deleted a base T at 388 bp downstream of ATG promoter. In addition, *osaai1-2* inserted a base A between 297 bp and 298 bp downstream of the promoter ATG, and *osaai1-3* inserted a base G between 840 bp and 841 bp downstream of the promoter ATG (Fig. 4A, Additional file 2: Fig. S4). The observation of *osaai1* mutant seedlings with normal growth for 14 days found that the phenotypes of the three mutant lines were the same (Additional file 2: Fig. S5A), so *osaai1-1* (hence to refer as *osaai1*) was selected for subsequent research and analysis.

We performed qRT-PCR on wild-type Zhonghua 11 (ZH11) and transgenic lines that grew for seven days to detect the expression of this gene in different lines. The results showed that the expression of this gene was significantly up-regulated in the overexpression line (OE19) compared to the ZH11, while it was significantly down-regulated in the mutant line (*osaai1*) due to early termination of transcription (Fig. 4B, Additional file 2: Fig. S5B, C). To further determine the biological function of *OsAAI1*, we performed statistical observation and data analysis on ZH11, *osaai1* and OE19 grown normally for 14 days. The results showed that *osaai1* showed dwarfing in plant height compared with ZH11 and OE19, and this difference may persist until later stages of whole development (Additional file 2: Fig. S6A–E). In addition, OE19 had better growth in primary root length, total root length, and adventitious root number than ZH11 and *osaai1* (Fig. 4C–G).

### Analysis of Agronomic Traits in *OsAAI1*

**Fig. 5:**
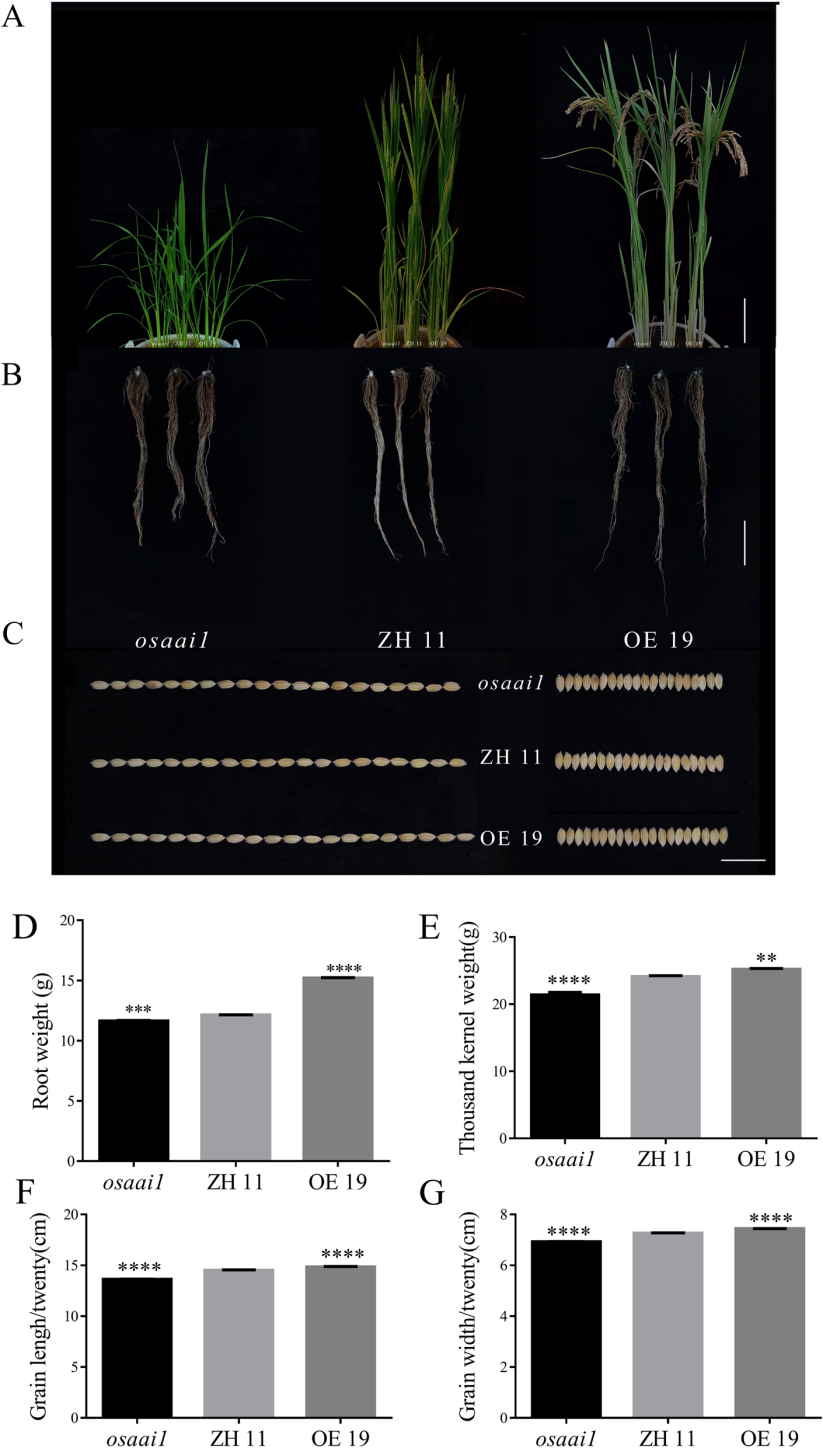
Analysis of agronomic traits in *OsAAI1*. **A** Phenotype diagram of *osaai1*, ZH11, and OE19 at tillering, heading and mature stage. Bars = 10 cm. **B** Phenotype diagram of root of *osaai1*, ZH11, and OE19 at mature stage. Bars = 10 cm. **C** Phenotype diagram and statistical analysis of grain length and grain width of *osaai1*, ZH11, and OE19, n = 20. Bars = 2.5 cm. **D** Statistical analysis of root weight, **E** thousand grain weight, **F** grain length and (G)grain width of each line at mature stage. (Asterisks indicate a statistically significant difference compared with ZH11. **P < 0.01, ***P < 0.001, ****P < 0.0001; One-way ANOVA).

To determine whether the dwarfed phenotype of *osaai1* would continue to affect the entire growth and development of rice. We further observed and statistically analyzed the plants at the tillering, heading and mature stage, and the results showed that plant height, leaf length and leaf width of *osaai1* decreased compared with ZH11 at the tillering stage, and conversely, these traits were increased significantly in OE19. At the heading stage, plant height of *osaai1* was still lower than ZH11, while the height of OE19 was not significantly difference compared with ZH11 (Fig. 5A, Additional file 2: Fig. S7A–C). Pollen viability of wild-type and transgenic lines at flowering period was stained to determine the pollen development of each line, and the results showed that the pollen staining rate of *osaai1* (44.33%) was only 65% of that of ZH11 (67.96%), while the staining rate of OE19 (90.30%) was 133% of that of ZH11 (Additional file 2: Fig. S7D, E). This indicates that *OsAAI1* may play an important role in rice pollen development. At mature stage, there were no significant differences between wild-type and transgenic lines in plant height, flag leaf length, top second leaf length, top third leaf length, flag leaf width, top second leaf width and top third leaf width, while the spike length of OE19 was significantly longer than that of ZH11 and *osaai1* (Additional file 2: Fig. S8A–C, Additional file 1: Table S2), whereas there were highly significant differences in seed setting rate, thousand grain weight, grain length, and grain width between wild-type and transgenic lines (Fig. 5C–G, Additional file 2: Fig. S7D, F, Additional file 2: Fig. S9A–C, Table 1), These results suggest that overexpression of *OsAAI1* improves rice yield through regulating the development of grain length and grain width. We weighed the fresh weight of the roots of each line at mature stage and found that the roots of OE19 were the heaviest and the longest (Fig. 5B). This indicates that *OsAAI1* gene not only regulates root development at the seedling stage but also at the mature stage. In summary, overexpression of *OsAAI1* significantly enhances rice yield by regulating root and grain development.

**Table 1:** Statistics of agronomic traits of wild-type and transgenic lines

Genotype	Pl	Tgw	Gl	Gw
*osaai1-1*	16.17****	22.31****	13.91****	6.42****
*osaai1-2*	17.30***	23.30**	13.22****	6.33****
*osaai1-3*	15.45****	23.03***	13.60****	6.64****
ZH 11	17.67	24.42	14.97	7.02
OE 6	18.15*	26.24****	15.71****	7.23****
OE 9	18.14*	25.67***	15.52****	7.31****
OE 18	18.93****	26.91****	15.65****	7.22****
OE 19	18.17*	25.27***	14.87****	7.44****

Pl = Panicle length (cm), n = 3. Tgw = Thousand grains weight (g), Gl = Grain length/twenty (cm), Gw = Grain width/twenty (cm), (Asterisks indicate a statistically significant difference compared with ZH11. **P < 0.01, ***P < 0.001, ****P < 0.0001; One-way ANOVA)

### *OsAAI1* Overexpression Improves Rice Tolerance to Drought Stress

**Fig. 6:**
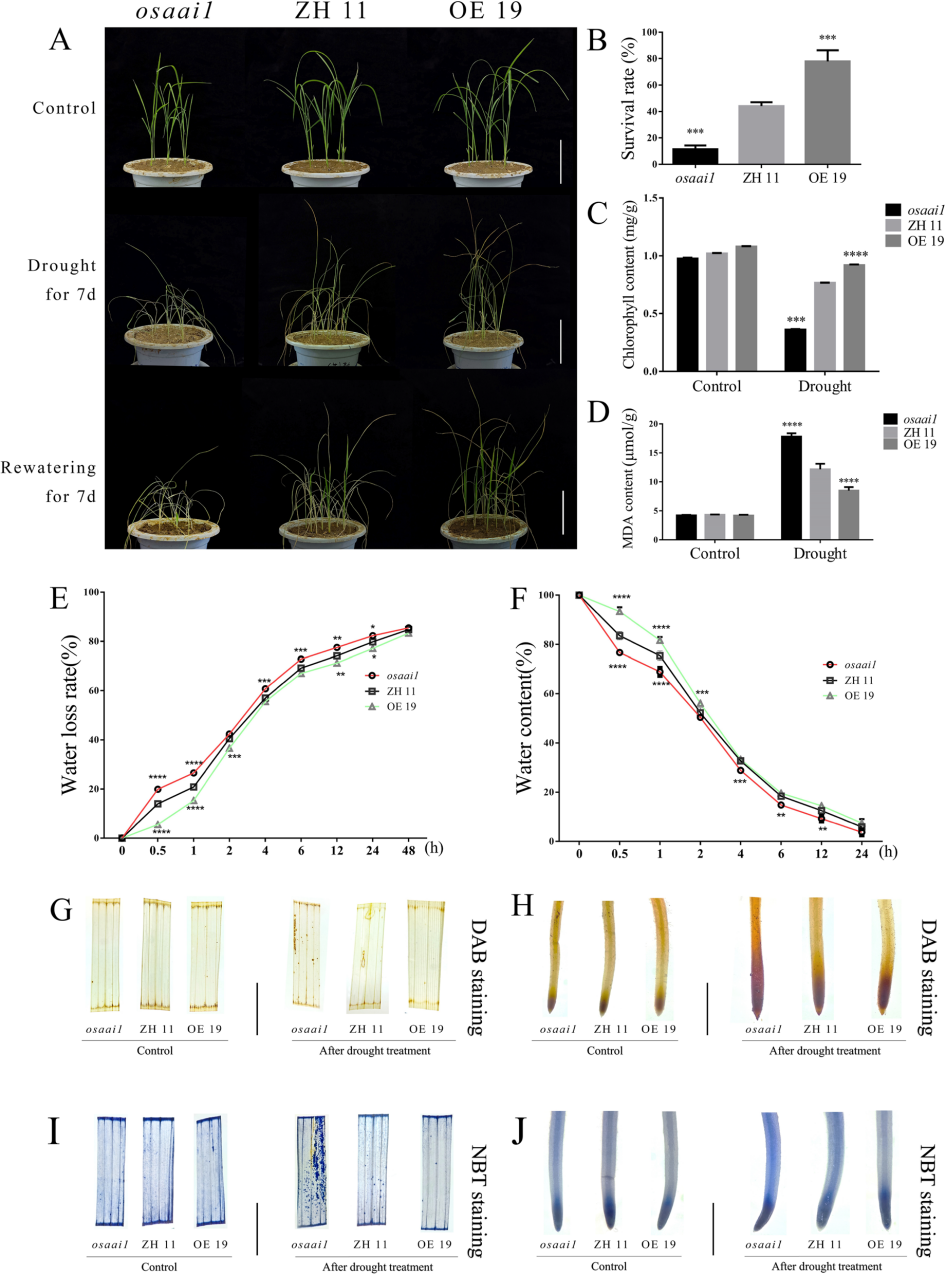
Phenotypes, physiological indicators and staining of each line under drought treatment. **A** Transgenic and wild-type plants were subjected to severe drought stress without water for 7d and then recovered for 7d, n = 9 Bars = 10 cm. **B** Survival rates, **C** chlorophyll content, **D** MDA content of *osaai1*, ZH11, and OE19 tested in A. **E** Comparison of water loss rates for detached rosettes between transgenic and wild-type plants (n = 5 plants). **F** Comparison of relative water contents of detached rosettes of transgenic and wild-type plants (n = 5 plants). **G-J** DAB and NBT staining of *osaai1*, ZH11, and OE19 tested in A. Bars = 1 cm. (Asterisks indicate a statistically significant difference compared with ZH11. **P < 0.01, ***P < 0.001, ****P < 0.0001; One-way ANOVA).

To verify the response of *OsAAI1* to drought stress, ZH11, *osaai1* and OE19 seedlings grown normally for 20 days were subjected to drought treatment, and after 7 days of dehydration and 7 days of water-rich recovery treatment (Fig. 6A), OE19 grew well and had higher survival rate (77.8%) compared with ZH11 (44.1%), whereas *osaai1* was severely inhibited in growth and the survival rate was low at 11.4% (Fig. 6B). Under normal growth, there was no significant difference in chlorophyll and MDA content between ZH11, *osaai1* and OE19, but after drought treatment, the difference in chlorophyll content between the wild-type and transgenic lines was highly significant, with higher chlorophyll content in OE19 and significantly lower chlorophyll content in *osaai1* compared with the drought-treated ZH11 (Fig. 6C). The MDA content was lower in OE19 and significantly higher in *osaai1* compared to the drought-treated ZH11 (Fig. 6D). We measured the dehydration rate and water content of wild-type and transgenic lines grown for 20 days and found that *osaai1* had the fastest water loss, followed by ZH11, and OE19 had the slowest water loss (Fig. 6E). Compared to ZH11 (water content was 5.88%), the final water content of *osaai1* was 3.69% and OE19 was 7.43% (Fig. 6F). These results suggest that *OsAAI1* plays a positive role in resistance to drought stress.

To investigate whether *OsAAI1* overexpression affects the accumulation and scavenging of ROS under drought stress, the levels of hydrogen peroxide (H_2_O_2_) and superoxide anion radical (O_2_^−^) were assessed by Diaminobezidin (DAB) and Nitroblue tetrazolium (NBT) staining. Under normal conditions, there was no significant difference in ROS accumulation between wild-type and transgenic lines. After drought treatment, a host of spots appeared on the *osaai1* line compared to ZH11, while OE19 staining was the lightest, indicating that *osaai1* accumulated a large amount of ROS, while the least amount of ROS was accumulated in OE19 (Fig. 6G–J). Relative quantification of ROS using imagej software and the results consistent with the above (Additional file 2: Fig. S10A–D, I–J). In conclusion, *OsAAI1* overexpression can enhance rice tolerance to drought stress and reduce ROS accumulation.

### Overexpression *OsAAI1* Improves Rice Tolerance to Osmotic Stress Tolerance

**Fig. 7:**
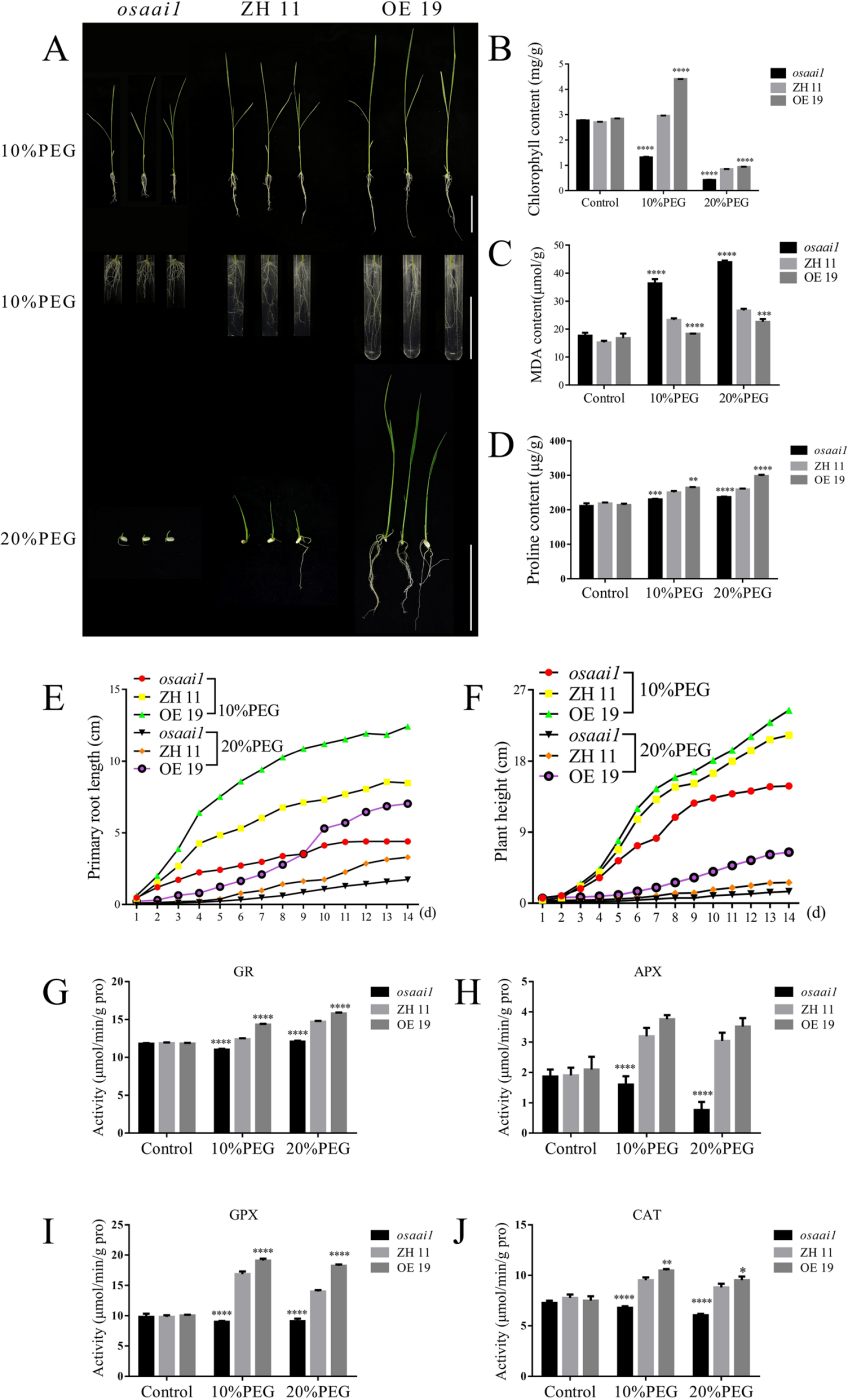
Phenotypic and physiological indices under different concentrations of PEG treatment. **A** Phenograms and root development of *osaai1*, ZH11, and OE19 treated with 10% and 20%PEG for 14 days. Bars = 5 cm. **B** Chlorophyll content of *osaai1*, ZH11, and OE19 tested in **A**. **C** MDA content of *osaai1*, ZH11, and OE19 tested in **A**. **D** Proline content of *osaai1*, ZH11, and OE19 tested in **A**. **E–H** ROS-related scavenger enzyme (CAT, APX, GPX, GR) activity assay tested in **A**. (Asterisks indicate a statistically significant difference compared with ZH11. **P < 0.01, ***P < 0.001, ****P < 0.0001; One-way ANOVA)

Osmotic stress is one of the main factors causing damage to plants under drought stress (Zhu 2016). To further verify the osmotic stress tolerance of *OsAAI1*, each rice line was treated with 10% and 20% Polyethylene glycol (PEG) 6000 (hence to refer as PEG) for 14 days to observe the phenotype and perform statistical analysis. The results showed that 10% PEG treatment promoted the root growth of OE19 and severely inhibited the root growth of *osaai1*, while the effect on ZH11 was not significant. Compared with the normal treatment, the primary roots of OE19 under 10% PEG treatment increased by an average of 2.55 cm and those of ZH11 increased by an average of 1.28 cm, while those of *osaai1* decreased by an average of 2.35 cm (Fig. 7A, E). The growth of the wild-type and transgenic lines was inhibited under 20% PEG treatment, with the mutant line being the most significantly inhibited and barely able to grow normally, OE19 showed the best growth compared to others (Fig. 7A, E, F). Physiological indicators were measured for each line treated with normal, 10% and 20% PEG for 14 days to assess the tolerance of wild-type and transgenic lines to different treatments. The chlorophyll content of ZH11, *osaai1* and OE19 did not significant difference under normal treatment, while the chlorophyll content of the *osaai1* was highly significantly reduced compared to ZH11 under 10% PEG treatment, while the chlorophyll content of OE19 was highly significantly increased, even higher than that under normal treatment. The chlorophyll content of both the wild-type and transgenic lines was significantly lower than that of the normal treatment under 20% PEG treatment, with *osaai1* having the lowest chlorophyll content and OE19 having the highest chlorophyll content (Fig. 7B). The MDA content of the wild-type and transgenic lines did not significant difference under normal treatment, but the MDA content of *osaai1* was significantly increased under both 10% PEG and 20% PEG treatment, while the MDA content of ZH11 was not significantly increased. It is noteworthy that the MDA content of OE19 showed a decreasing trend under 10% PEG treatment, and was even lower than the normal, although the MDA content of OE19 also showed an increase under 20% PEG treatment, it was always lower than that of ZH11 and *osaai1* under the same treatment (Fig. 7C). There was no significant difference in proline content between wild-type and transgenic lines under normal treatment, and proline content was significantly higher in both wild-type and transgenic lines under 10% PEG and 20% PEG treatments, but OE19 accumulated the most proline in both 10% PEG and 20% PEG treatments (Fig. 7D). We further examined the changes of ROS-related scavenger enzymes (such as CAT, APX, GPX, GR) activities under each treatment, as shown in the Fig. 7G–J, the ROS-related scavenger enzymes activities of OE19 were significantly enhanced under 10% and 20% PEG treatment, while the ROS-related scavenger enzymes activities of *osaai1* were significantly weakened. In conclusion, *OsAAI1* overexpression can enhance rice tolerance to drought stress and osmotic stress by enhancing ROS scavenging ability.

### *OsAAI1* is Involved in the ABA-Mediated Regulatory Pathway

**Fig. 8:**
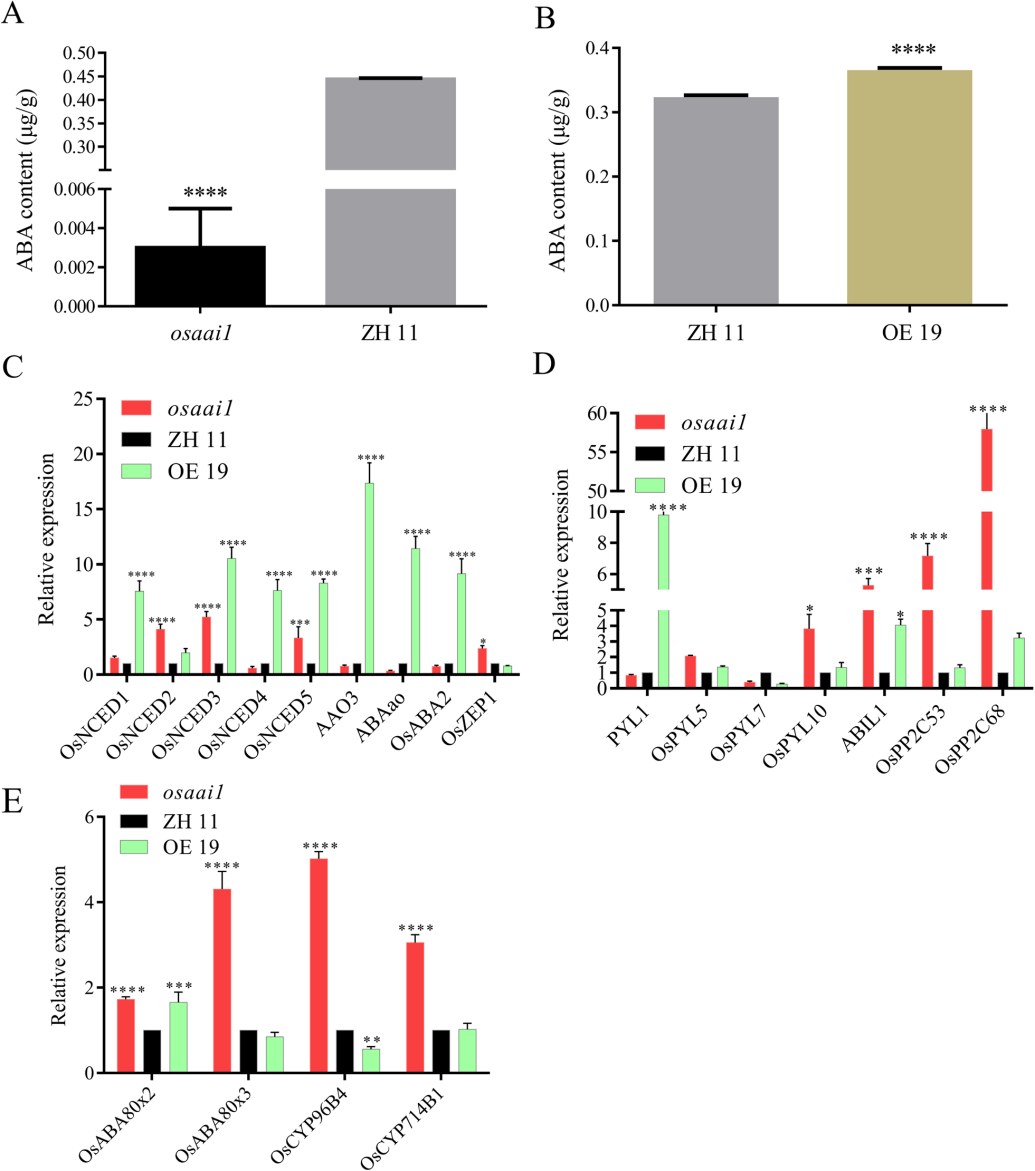
*OsAAI1* is involved in the ABA signaling pathway. **A** ABA content of *osaai1*, ZH11, and OE19. (**P < 0.01, ***P < 0.001, ****P < 0.0001, *t-test*) **B** qRT-RCR of ABA biosynthesis-related genes in *osaai1*, ZH11, and OE19. (**P < 0.01, ***P < 0.001, ****P < 0.0001, *t*-test) **C** qRT-RCR of ABA signal transduction-related genes in *osaai1*, ZH11, and OE19. **D** qRT-RCR of ABA catabolism-related genes in *osaai1*, ZH11, and OE19. (Asterisks indicate a statistically significant difference compared with ZH11. **P < 0.01, ***P < 0.001, ****P < 0.0001; One-way ANOVA)

To further verify whether the drought resistance of *OsAAI1* was induced by ABA, we measured the ABA content of the wild-type and transgenic lines. The results showed that *osaai1* had extremely lower ABA content and OE19 had significantly higher ABA content than ZH11(Fig. 8A, B). Many genes are involved in ABA biosynthesis in rice, such as 9-cis-epoxycarotenoid dioxygenase1-5(*OsNCED1*–*5*) (Parry et al. 1990), abscisic aldehyde oxidse3 (*AAO3*), abscisic acid2 (*ABA2*), and zeaxanthin epoxidase1 (*OsZEP1*) (Schwartz et al. 1997; Seo et al. 2000). pyrabactin resistance 1-like5 (*OsPLY5*), *PYL1, OsPLY7*, *OsPLY10* (Bhatnagar et al. 2020; Di F et al. 2018; Kim et al. 2014; Verma et al. 2019), protein phosphatase2c53 (*OsPP2C53*), *OsPP2C68* (Min et al. 2019; Xiong et al. 2014). Cytochrome p450 96b4 (*OsCYP96B4*), *OsCYP714B1*, aba 8′-hydroxylase2 (*OsABA8ox2*) and *OsABA8ox3* are vital for ABA degradation (Cai et al. 2015; Magome et al. 2013; Mega et al. 2015; Ramamoorthy et al. 2011). The transcript levels of different ABA related genes were examined by qRT-PCR in *osaai1*, OE19 and ZH11 lines. The crucial rate-limiting enzyme family genes for ABA biosynthesis (*OsNCED1*–*5*) were found to be significantly up-regulated in OE19, and the positive ABA biosynthesis-related genes *AAO3*, *ABAao*, and *OsABA2* were down-regulated in *osaai1* and up-regulated in OE19 compared with those in the ZH11(Fig. 8C). The ABA receptors *OsPYL5* and *OsPYL10* were both up-regulated in the transgenic lines and more significantly in *osaai1*, *PYL1* expression was down-regulated in *osaai1* and up-regulated in OE19, while the transcript levels of *OsPYL7* was not obviously changed in *osaai1* and OE19 (Fig. 8D). *OsPP2C53* and *OsPP2C68*, negative regulators of ABA signaling, were most significantly up-regulated in *osaai1* (Fig. 8D). ABA degradation-related genes *OsCYP96B4, OsCYP714B1* and *OsABA8ox3* expression was up-regulated in *osaai1* and down-regulated in OE19, While the transcript levels of *OsABA8ox2* was not obviously changed in *osaai1* and OE19 (Fig. 8E). This indicates that *OsAAI1* is involved in ABA biosynthesis, catabolism and signaling processes. Taken together, *OsAAI1* responds to drought stress by inducing the expression of genes related to ABA biosynthesis, catabolism and signaling, indicating that *OsAAI1* is depend on ABA pathway to improve tolerance to drought stress in rice.

### Overexpression of *OsAAI1* is Sensitive to ABA

**Fig. 9:**
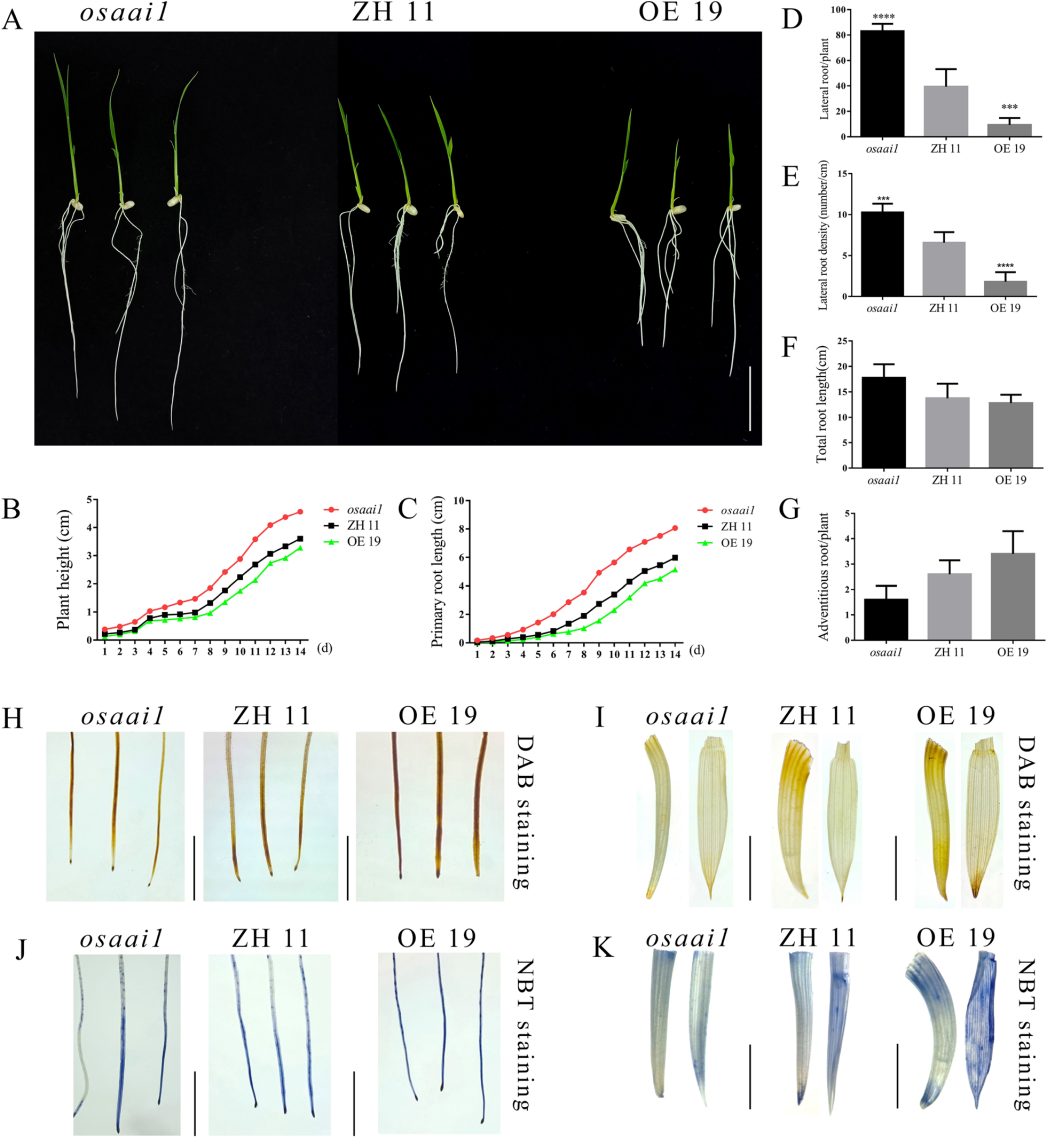
Overexpression of *OsAAI1* is sensitive to ABA. **A–G** Phenotypic and data statistical analysis of *osaai1*, ZH11, and OE19 treated with 3 µM ABA for 14 days. Bars = 2.5 cm. **H–K** DAB and NBT staining of *osaai1*, ZH11, and OE19 tested in A. Bars = 0.1 cm. (*t-test*, **P < 0.01, ***P < 0.001, ****P < 0.0001)

To investigate the sensitivity of *OsAAI1* to ABA, we statistically analyzed the growth status of wild-type and transgenic lines at 14 days of 3 µM ABA treatment. Under normal conditions, OE19 grew significantly better than *osaai1* and ZH11, while under 3 µM ABA treatment, the plant height and primary root length of OE19 were significantly lower than those of ZH11 and *osaai1*, and *osaai1* grew the best among all strains (Fig. 4C–G; Fig. 9A–G). We performed DAB and NBT staining of each line after 14 days of 3 µM ABA treatment to assess the ROS accumulation in each line. The results showed that OE19 of DAB and NBT staining was the darkest, followed by ZH11, and *osaai1* was the lightest (Fig. 9H–K), indicating that more ROS accumulated in OE19. Relative quantification of ROS using imagej software and the results consistent with the above (Additional file 2: Fig. S10E–H, K–L).These results suggest that overexpression of *OsAAI1* enhanced the sensitivity to ABA.

## Discussion

### The Biological Function and Molecular Mechanism of the HPS_like Subfamily

Multiple sequence alignments and phylogenetic analysis indicated that *OsAAI1* belongs to the HPS_like subfamily of the AAI_LTSS superfamily. Members of this gene family encode three domains including LTP2 domain, hydrophobic seed domain, and trypsin alpha amylase domain (Lu et al. 2020; Qanmber et al. 2019). The subfamily HPS_like is composed of HPS, a dark-inducible protein (*LeDI-2*) from *Lithospermum erythrorhizon* (Yazaki et al. 2001), a hybrid proline-rich protein (*HyPRP*) from maize (Jose-Estanyol et al. 1992), rice *RcC3* protein (Xu et al. 1995), and maize *ZRP3* protein (John et al. 1992). There are a total of 22 AAI genes in the rice genome, among which only two genes had been reported (Additional file 1: Table S1). *RCc3* (LOC_Os02g44310), which enhances salt tolerance in rice by regulating root structure (Xu et al. 1995), and *qLTG3–1 *(LOC_Os03g01320) which involves in seed germination and show tolerance to several kinds of abiotic stresses (Fujino et al. 2008), but both of them specific molecular mechanism is unclear. The specific biological functions of the remaining 20 AAI genes have not been explored (Additional file 1: Table S1). In this study, we report a rice AAI gene, *OsAAI1* (LOC_Os04g55159), overexpression of which improves rice root and shoot development and regulates rice grain size development (Figs. 4C, E, 5B–D, Additional file 2: Fig. S9A–C, Table 1), thereby enhancing rice yield. However, the molecular mechanism of *OsAAI1*, that needs to be further studied.

### *OsAAI1* Confers Drought Tolerance Dependent on ABA-Mediated Regulatory and ROS Scavenging Pathway

The plant hormone ABA plays a crucial role in the plant's response to environmental stress (Fujita et al. 2011; Gietler et al. 2020). There is an optimal level of ABA for root elongation (Zhang et al. 2014), l ABA concentration above a certain value will inhibit plant growth (Miao et al. 2021). For the result that OE19 had higher plant height and longer primary root length than ZH11 and *osaai1* in seedling stage (Fig. 4C–G), this can be explained by the fact that the ABA content of OE19, although significantly higher than ZH11 and *osaai1* (Fig. 8A, B), was well below the threshold of ABA concentration that inhibits plant growth, and therefore the growth of OE19 was not inhibited. And for the result that OE19 have increased sensitivity to exogenous ABA (Fig. 9A–G), this can be explained by the fact that the addition of exogenous ABA to OE19 exceeded the optimal concentration of ABA for plant growth, and the addition of ABA can be considered as a stress treatment that moves them further away from this optimal concentration. In contrast, ZH11 and *osaai1* may have experienced a lower ABA concentration and the addition of ABA would have caused them less harm, which is consistent with the previous studies (Chen et al. 2018; Jiang et al. 2019; Jiang et al. 2018). Overexpression of *OsAAI1* increases the sensitivity of rice seedlings to exogenous ABA and increases the content of endogenous ABA (Fig. 8A, B Fig. 9A–G), indicating that *OsAAI1* is involved in the ABA-mediated regulatory pathway. ABA biosynthesis is further converted from mevalonate by carotenoids (Dong et al. 2015), NCED is a key rate-limiting enzyme for ABA biosynthesis (Parry et al. 1990), and NCED was first identified in the maize *vp14* mutant (Tan et al. 1997). *OsNCED1* overexpression in rice enhances tolerance to heat stress during tasseling and flowering by increasing antioxidant capacity (Zhou et al. 2022). Overexpression of *BnNCED3* in oilseed rape promoted NO and ROS production in transgenic *Arabidopsis thaliana* and increased ABA accumulation thereby enhancing abiotic stress tolerance in *A. thaliana* (Xu and Cai 2017). In the present study, the key genes for ABA biosynthesis, *OsNCED1*, *OsNCED2, OsNCED3, and OsNCED5*, were up-regulated in the transgenic lines and were most significantly up-regulated in the overexpression lines (Fig. 8C). In addition to NCED family genes, *AAO3*, *ABAao*, and *OsABA2* also play active roles in ABA biosynthesis (Schwartz et al. 1997; Seo et al. 2000). The positive ABA biosynthesis-related genes *AAO3*, *ABAao*, and *OsABA2* were found to be significantly upregulated in OE19 (Fig. 8C). In the ABA signaling pathway, *OsPYL/RCAR* is a functional ABA receptor that regulates ABA-dependent gene expression in rice (Bhatnagar et al. 2020; Di F et al. 2018; Kim et al. 2014; Verma et al. 2019). In this study, *PYL1* expression was down-regulated in *osaai1*, while significantly up-regulated in OE19 (Fig. 8D). In ABA catabolism, 8' hydroxylation of ABA is the main mode of ABA degradation in plants (Kushiro et al. 2004), and *OsABA8ox3* is a key gene in ABA catabolism (Cai et al. 2015). ABA degradation-related genes *OsCYP96B4, OsCYP714B1 and OsABA8ox3* expression was up-regulated in *osaai1* and down-regulated in OE19 (Fig. 8E). Type 2C protein phosphatase (PP2C) is a negative regulator in ABA signaling (Li et al. 2015). PP2C inhibits SnRK2s activity through dephosphorylation, resulting in inhibition of ABA response element binding proteins (AREBs) and ABA response element binding factors (ABFs) downstream of SnRK2s (Ma et al. 2009), thereby suppressing the expression of ABA-related genes (Min et al. 2019). The negative regulators of ABA signaling *ABIL1*, *OsPP2C53* and *OsPP2C68* were most significantly up-regulated in *osaai1* (Fig. 8D), showing that *OsAAI1* increases the tolerance of rice under drought stress by regulating the expression of ABA-mediated genes. These results suggest that *OsAAI1* plays a crucial role in drought stress via the ABA-mediated regulatory pathway.

Reactive oxygen species are key signal transduction molecules in plants, but their excessive accumulation can cause irreversible damage to cells (Apel and Hirt 2004; Mittler et al. 2011; Tanaka et al. 2006; Torres and Dangl 2005). Previous studies have shown that plants adapt to abiotic stresses by regulating ROS metabolism (Fang et al. 2015; Schmidt et al. 2013; Wu et al. 2012). For example, overexpression of MAPK kinase *DSM1* in rice improves drought tolerance in rice at the seedling stage by regulating ROS clearance in rice (Ning et al. 2010). the NAC transcription factor *NTL4* enhances ROS accumulation in response to drought stress by binding to the promoter of the gene encoding ROS biosynthetic enzyme (Lee et al. 2012). Recent studies (Aleem et al. 2022; Kuo et al. 2020; Wu et al. 2015; Xu et al. 2018) indicated that increased APX, GR, CAT and GPX activities can improve ROS clearance and maintain ROS homeostasis, thereby improving environmental-stress tolerance. In the present study, overexpression of *OsAAI1* increased the activity of the ROS scavenging enzymes CAT, APX, GPX and GR in rice under osmotic stress. DAB and NBT staining results indicated that *OsAAI1* overexpression accumulated less O_2_^−^ and H_2_O_2_ under drought stress than the wild type (Fig. 6G–J, Fig. 7G–J). These results indicate that *OsAAI1* can reduce ROS accumulation under drought and osmotic stress. To sum up, these results showed that the functions of *OsAAI1* in drought tolerance might be associated with the regulation of antioxidation ability.

## Drought Stress Interact with ABA-Mediated Regulatory and ROS Scavenging Pathway

In this study, we showed that *OsAAI1* regulated root tip and leaf ROS levels (Fig. 6G–J, Fig. 9H–K) and altered ABA sensitivity in rice (Fig. 9A–G), which suggests that ROS alteration is correlated to ABA sensitivity. It has been reported that alterations in ROS levels can affect ABA biosynthesis and signaling, as well as change ABA sensitivity (Chen et al. 2020; Postiglione and Muday 2020; Song et al. 2014), and ABA can also regulate the expression of ROS producing and scavenging genes (Chen et al. 2022a, b; Yu et al. 2019). For instance, a link between ABA signaling and H_2_O_2_ production via G-proteins that are shown to promote H_2_O_2_ production but negatively regulate ABA response had been proved (Chen et al. 2004; Xu et al. 2015). These data suggest that there are likely to be different mechanisms by which ABA signaling and ROS production interact and regulate each other. All of these observations strengthen the link between the changed ROS levels and altered ABA response in *OsAAI1* transgenic plants (Fig. 9A–K). In many cases, abiotic stress gives rise to various metabolic changes, known as elevated ROS levels (Hasanuzzaman et al. 2020; Qiu et al. 2021). Along with increased ROS, ABA signaling and ABA-dependent proline accumulation, have been proposed to be crucial components of cross tolerance to various stresses (Cao et al. 2020; Chen et al. 2022a, b; Xu et al. 2018).

## Conclusions

Bioinformatics analysis reveals that *OsAAI1* belongs to the HPS_like subfamily of the AAI_LTSS superfamily. Overexpression *OsAAI1* significantly increased rice roots and aboveground parts growth and development, thereby enhances the yield of rice. Overexpression *OsAAI1* inducing the expression of genes related to ABA biosynthesis, catabolism and signaling pathway and increasing the activities of many antioxidant defense enzymes of maintain the ROS balance to improve tolerance to drought stress in rice. In summary, *OsAAI1* not only increases the yield of rice but also improve it drought stress tolerance with the ABA-mediated regulatory and ROS scavenging pathway.

## Materials and Methods

### Bioinformatics Analysis of *OsAAI1*

The protein sequence of OsAAI1 was homologously sequence aligned in NCBI (www.ncbi.nlm.nih.gov), and phylogenetic trees between OsAAI1 and its homologous sequences were constructed in MEGA7.0 (adjacency method NJ and p-distance calculation method, bootstrap was set to 1000, other settings are default). Motif prediction analysis was performed in online website MEME (https://meme-suite.org/meme/doc/meme.html) (the number of conservative motif retrieval was set to 10). Combine the motif of the phylogenetic tree and MEME prediction into TBtools. GeneDOC was used to compare OsAAI1 with *Arabidopsis* and rice homologous protein sequences.

*OsAAI1* transcriptional prediction analysis: http://glab.hzau.edu.cn/RiceENCODE/;

OsAAI1 protein prediction analysis: https://web.expasy.org/protparam/;

*OsAAI1* expression prediction analysis: http://bar.utoronto.ca/efp2/;

*OsAAI1* promoter analysis: http://bioinformatics.psb.ugent.be/webtools/plantcare/html.

### Plant Materials and Hormone Treatment

The Clustered regularly interspaced short palindromic repeats-associated protein-9 (CRISPR-Cas9) gene editing vector pYLCRISPR-Cas9Pubi-T1 was constructed (ggccaacatcctca). Mutant lines (*osaai1-1*, *osaai1-2*, *osaai1-3*) were obtained by genetic transformation on the basis of *Oryza Sativa* L. (Zhong Hua 11). Overexpression lines (OE2, OE3, OE6, OE9, OE18, OE19) were constructed into overexpression vector pEGOEP35s-A-GUS on the basis of *Oryza Sativa* L. (Zhong Hua 11). After disinfection, seeds germinated at 30 °C for 2–3 days for phenotypic analysis. When the buds grow to about 1 mm, inoculate them in Murashige and Skoog (1/2 MS) medium, cultivate them in an artificial climate incubator at 30 °C, 16 hours light / 8 hours dark, observe and statistically analyze each lines phenotype. Drought stress analysis of transgenic plants based on the above methods. The early seedlings (bud length of about 1 mm) were placed in 10% and 20% PEG6000 solution for 14 days, and the phenotype data was measured. Finally, the seedlings which normally grew for 14 days were cultured in sandy soil; then, watering was stopped for 7 days to simulate field drought until the leaves curled, followed by recovery with normal watering for another 7 days to calculate the survival rate. Sensitivity analysis of ABA was based of the above method, ABA was added to (1/2 MS) medium at around 40 °C to make the final concentration 3 µM. Then, observation and statistical analysis. Phenotype of germ lines after hormone treatment, hormone and abiotic stress treatments were performed on ZH11 seedlings grown for 7 days in 1/2 MS medium at 4 °C and 42 °C, respectively, in final concentrations of 100 µM ABA, 10 nM IAA, 1 mM H_2_O_2_, 20%PEG, and 100 mM mannitol, and sampling at 0, 4, 8, 24, and 48 h. The specific operation of the dehydration treatment was that ZH11 grown normally for 7 days was pulled directly from the medium, the roots were rinsed and dried, and left at 25 °C for 48 h. Samples were taken for qRT-PCR experiments at 0, 4, 8, 24, and 48 h, respectively.

### Total RNA Extraction and Quantitative PCR Analysis

Total RNA was extracted using Life (code: 15596-026) Trizol reagent. RNA was used to synthesize cDNA using a HiScript II qRT SuperMix for qPCR (+ gDNA wiper) reagent Kit from Vazyme (code: R223-01). qRT-PCR was performed on Bio-Rad CFX96 instrument with ChamQ Universal SYBR qPCR Master Mix reagent (Vazyme: Q711-02) according to manufacturer’s instructions. The gene *β-Actin* as rice housekeeping gene was used for an internal reference. Each analysis includes three biological repeats and three technical replicates. Primers used for qRT-PCR are listed in Additional file 1: Table S3.

### Subcellular Localization

The CDS sequence of *OsAAI1* was cloned into the pCAMBIA1301 GFP vector, and the plasmid was transformed into rice protoplast isolated from rice suspension culture cells using PEG4000 mediated protoplast transformation (Shen et al. 2014; Yoo et al. 2007). The protoplasts were then transferred to a porous plate and cultured in the dark at room temperature for 16–20 h. Carl Zeiss axioskop 2 confocal microscope and the image acquisition software Zen Blue Edition (Carl Zeiss, Oberkochen, Germany) were used to observe the green fluorescence signal of transfected protoplasts. The empty GFP plasmid was used as a control. GFP fluorescence was detected at 488 nm excitation and 561 nm emission wavelength. Primers used for these constructs are listed in Additional file 1: Table S4.

### Quantification of Endogenous ABA

For ABA quantification, the plants of two-weeks-old ZH11, OE19 and *osaai1* were harvested and used for measurement of ABA by a liquid chromatography system (ACCHROM S3000) high performance liquid chromatograph, Alphasil VC-C18 (4.6 mm * 250 mm, 5 µm) chromatography measure the contents of ABA.

### Analysis of Pollen Viability

To observe starch accumulation, the pollen grains from the transgenic as well as the wild-type plants were collected and stained with 1% iodine-potassium iodide solution. Then the pollen grains were directly examined under a stereo microscope. The deeply stained and round pollen grains were counted as viable and pictures were taken.

### Water Loss Rate Determination

Water loss rates were measured using five plants each of wild-type and transgenic plants. Four-week-old plants were detached from roots and weighed immediately (fresh weight, FW), then the plants were left on the laboratory bench (humidity, 45–50%, 20–22 °C) and weighed at the designated time intervals (1 h, 2 h, 4 h, 6 h, 12 h, 24 h). The proportions of fresh weight loss were calculated relative to the initial plant weight. The plants were finally oven dried for 24 h at 80 °C to a constant dry weight (DW). Relative water contents (RWCs) and water loss rate were measured according to the formula: Water loss rate (%) = (FW– dry weight)/FW × 100), RWC (%) = (desiccated weight–DW)/(FW–DW) ×100.

### Physiological Measurements

Leaves from plants exposed to drought stress (including natural drought, 10% PEG6000 and 20% PEG6000 treatment) for 14 days were used to measure physiological index, and plants grown in normal conditions were used as control. Total chlorophyll content was determined by the protocol as described previously (Huang et al. 2009). MDA content was determined as previously described (Tang et al. 2013). Free proline content was measured using the reported method (He et al. 2012). Fresh leaf samples were used for enzyme extraction. All operations were carried out at 4 °C. CAT, APX, GPX, GR activity was measured according to the method as described previously (Aebi 1984; Murshed et al. 2008). Superoxide anion radical and hydrogen peroxide accumulation were detected by NBT and DAB staining. The plants samples were excised and immediately placed in 50 mM sodium phosphate buffer (pH 7.5) containing 1 mg/mL NBT at 28 °C for 8 h in the dark. Leaves were placed in 1 mg/mL DAB solution (pH 3.8) and incubated at 28 °C for 12 h in the dark. 90% ethanol and anhydrous ethanol was used to remove chlorophyll. The accumulation of hydrogen peroxide and superoxide anion radical was observed under a stereo microscope.

### Relative Quantification of ROS

Relative quantitative analysis of ROS was analyzed according to the method as described previously (Juszczak and Baier 2014).

### Data Analysis

The relative expression of genes was analyzed using the 2^−ΔΔCt^ method, and statistical analysis was performed in GraphPad prism 6.0 software. For phenotypic data analysis, GraphPad prism 6.0 software was used for statistical analysis.

### Supplementary Information

**Additional file 1: Table S1.** Prediction of amino acid sequence information and subcellular localization of homologous genes of* OsAAI1* in* Oryza sativa* L. and* Arabidopsis thaliana* L.** Table S2.** Trait statistics of each line at maturity.** Table S3.** qRT-PCR primers for ABA-related genes and* OsAAI1*.** Table S4.** Primer sequences.

**Additional file 2: Fig. S1.** Bioinformatics Analysis process of* OsAAI1*.** Fig. S2.** Bioinformatics Analysis of* OsAAI1*.** Fig. S3.** Bioinformatic predictive analysis of* OsAAI1*.** Fig. S4.** Sequencing alignment analysis of* osaai1* mutation sites.** Fig. S5.** Phenotypic analysis of transgenic strains at seedling stage.** Fig. S6.** Comparison and statistical analysis between wild-type and transgenic lines at flowering stage.** Fig. S7.** Pollen viability staining of ZH11,* osaai1* and OE19 at flowering.** Fig. S8.** Analysis of agronomic traits of each line.** Fig. S9.** Comparison of grain size between wild type and transgenic lines.** Fig. S10.** Relative quantitative analysis of ROS.

## Data Availability

All of the datasets are included within the article and its additional files.

## Abbreviations

ABA: Abscisic acid
ROS: Reactive oxygen species
qRT-PCR: Real time quantitative polymerase chain reaction
GFP: Green Fluorescent Protein
ZH11: Zhong Hua 11
OE: Overexpression
DAB: Diaminobezidin
NBT: Nitroblue tetrazolium
PEG6000: Polyethylene glycol
CRISPR-Cas9: Clustered regularly interspaced short palindromic repeats-associated protein-9
