# Supplementary material for: OsAAI1 Increases Rice Yield and Drought Tolerance Dependent on ABA-Mediated Regulatory and ROS Scavenging Pathway
Source: Rice (N Y). 2023 Aug 3;16:35. doi: 10.1186/s12284-023-00650-3 (PMC10400514; doi:10.1186/s12284-023-00650-3)
Supplement: Supplementary file 2 — Additional file 2: Fig. S1. Bioinformatics Analysis process of OsAAI1. Fig. S2. Bioinformatics Analysis of OsAAI1. Fig. S3. Bioinformatic predictive analysis of OsAAI1. Fig. S4. Sequencing alignment analysis of osaai1 mutation sites. Fig. S5. Phenotypic analysis of transgenic strains at seedling stage. Fig. S6. Comparison and statistical analysis between wild-type and transgenic lines at flowering stage. Fig. S7. Pollen viability staining of ZH11, osaai1 and OE19 at flowering. Fig. S8. Analysis of agronomic traits of each line. Fig. S9. Comparison of grain size between wild type and transgenic lines. Fig. S10. Relative quantitative analysis of ROS. [file 12284_2023_650_MOESM2_ESM.pptx]

## Slide 1
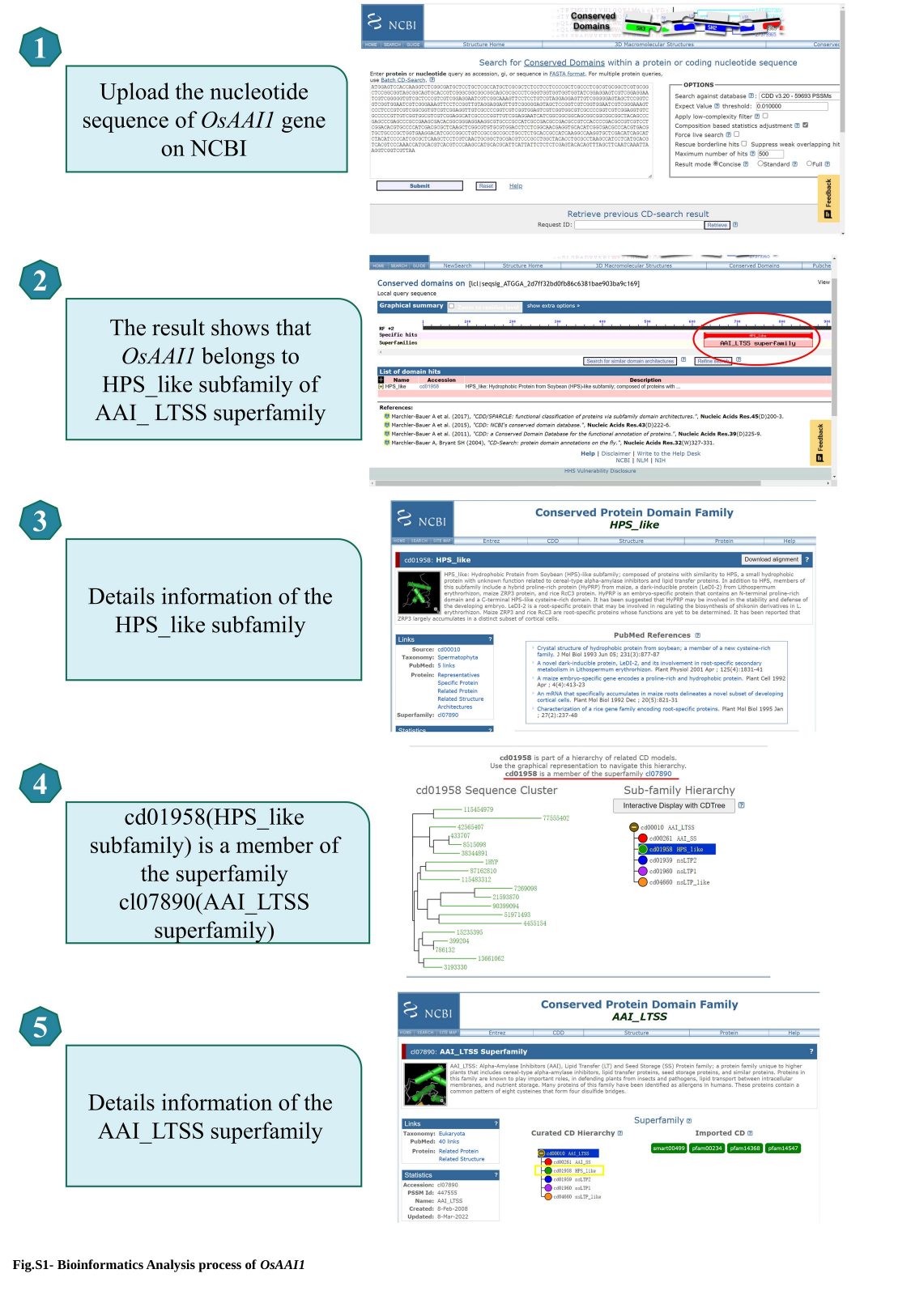

Fig.S1- Bioinformatics Analysis process of OsAAI1

## Slide 2
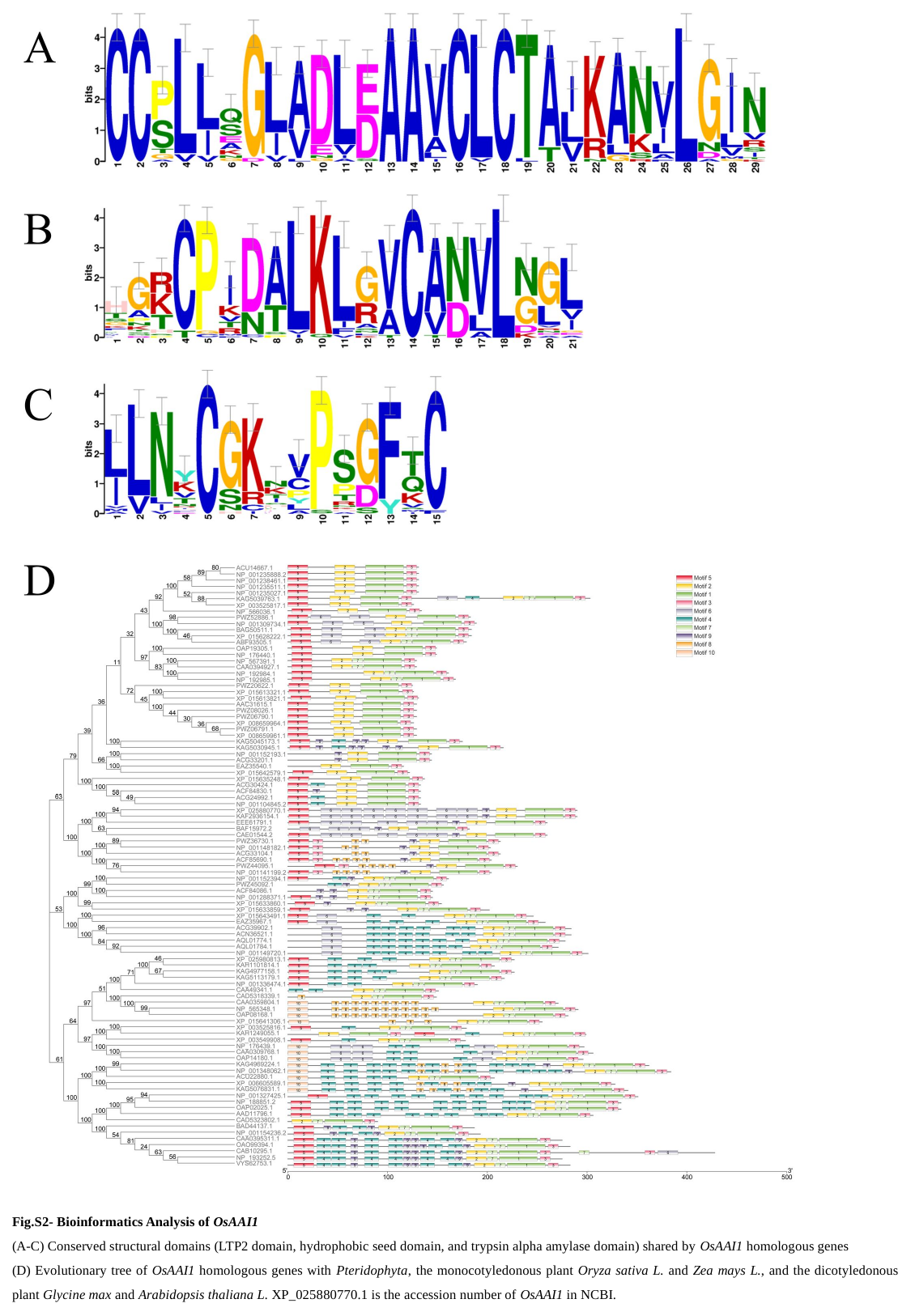

Fig.S2- Bioinformatics Analysis of OsAAI1
(A-C) Conserved structural domains (LTP2 domain, hydrophobic seed domain, and trypsin alpha amylase domain) shared by OsAAI1 homologous genes
(D) Evolutionary tree of OsAAI1 homologous genes with Pteridophyta, the monocotyledonous plant Oryza sativa L. and Zea mays L., and the dicotyledonous plant Glycine max and Arabidopsis thaliana L. XP_025880770.1 is the accession number of OsAAI1 in NCBI.

## Slide 3
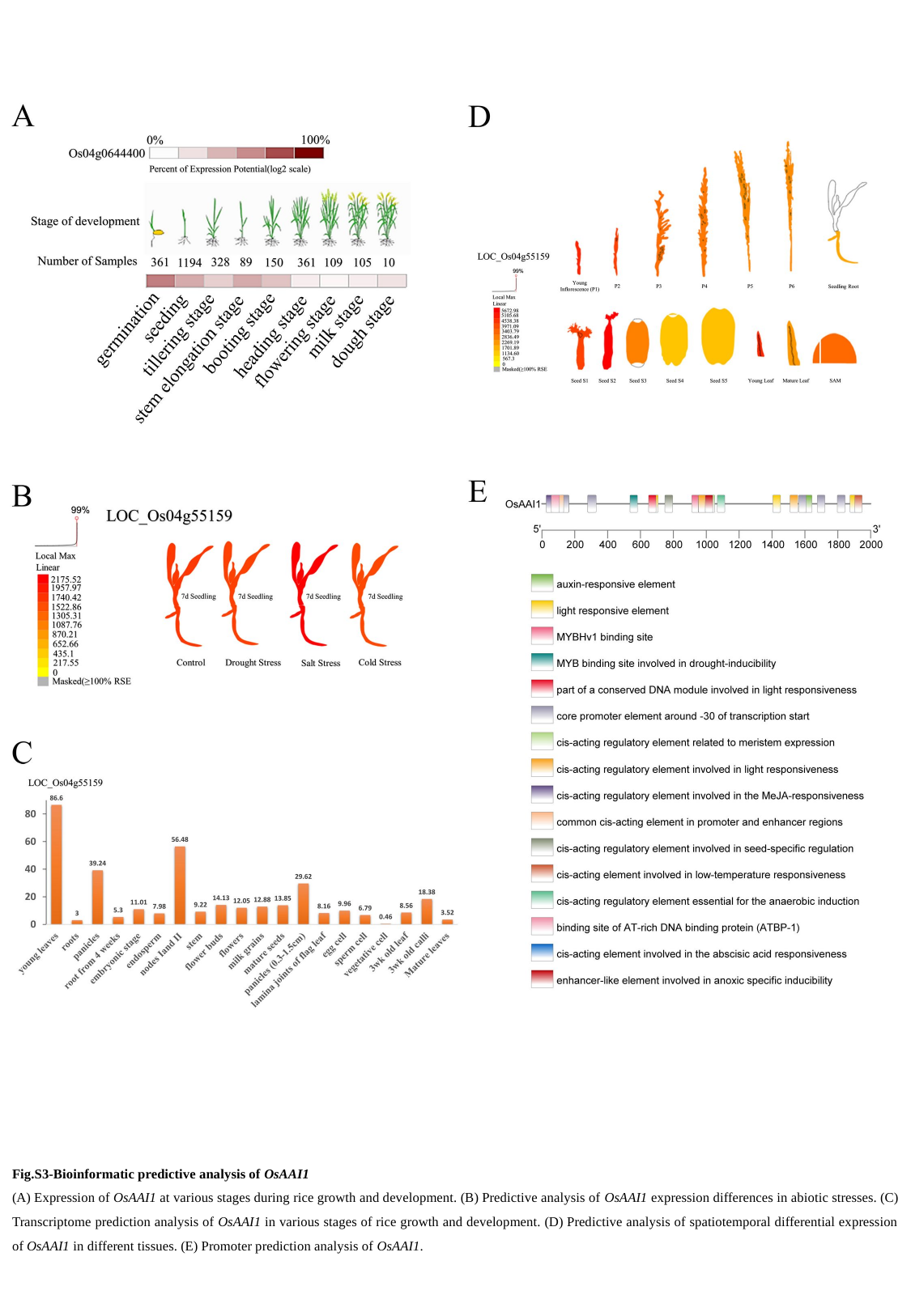

Fig.S3-Bioinformatic predictive analysis of OsAAI1
(A) Expression of OsAAI1 at various stages during rice growth and development. (B) Predictive analysis of OsAAI1 expression differences in abiotic stresses. (C) Transcriptome prediction analysis of OsAAI1 in various stages of rice growth and development. (D) Predictive analysis of spatiotemporal differential expression of OsAAI1 in different tissues. (E) Promoter prediction analysis of OsAAI1.

## Slide 4
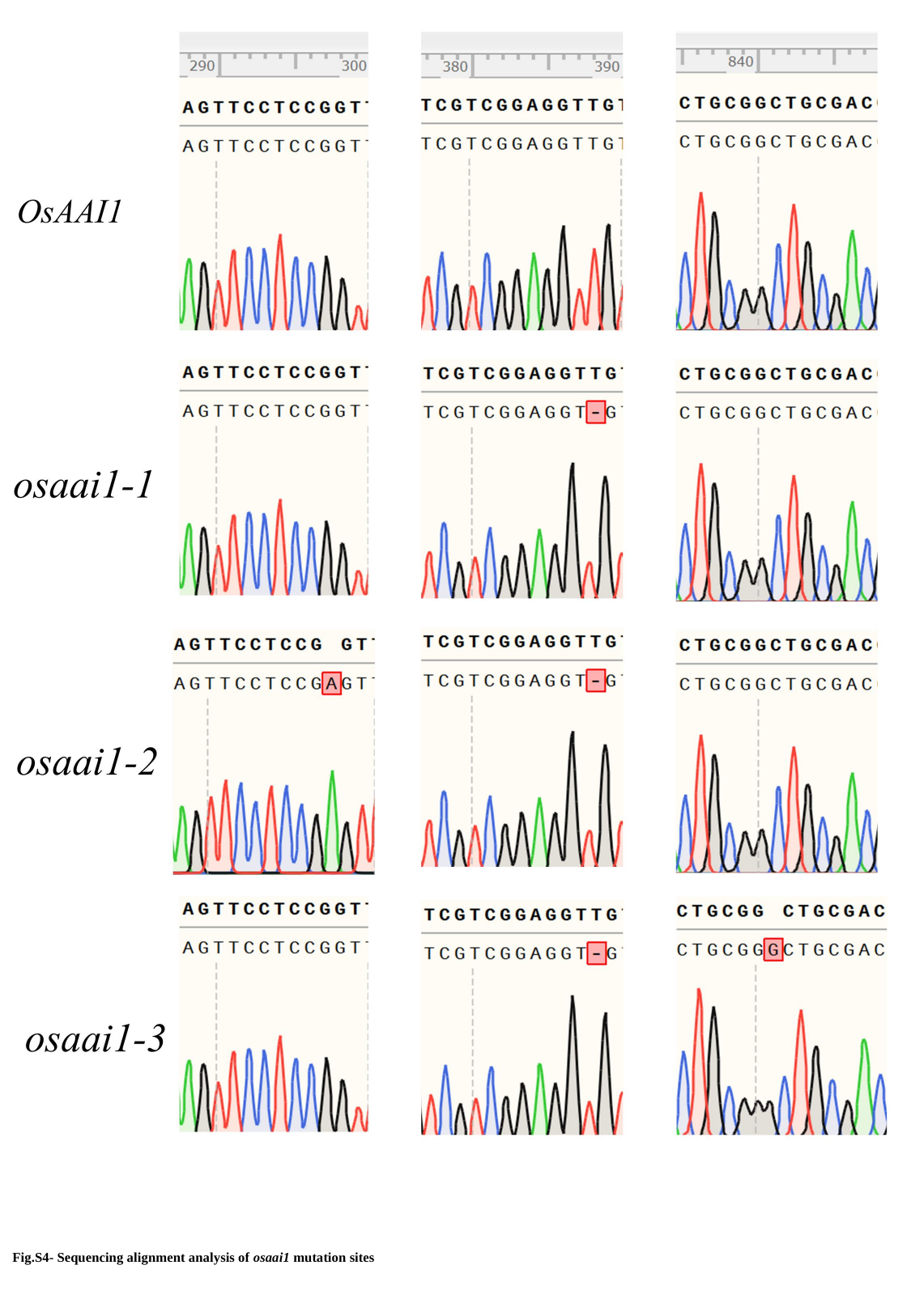

Fig.S4- Sequencing alignment analysis of osaai1 mutation sites

## Slide 5
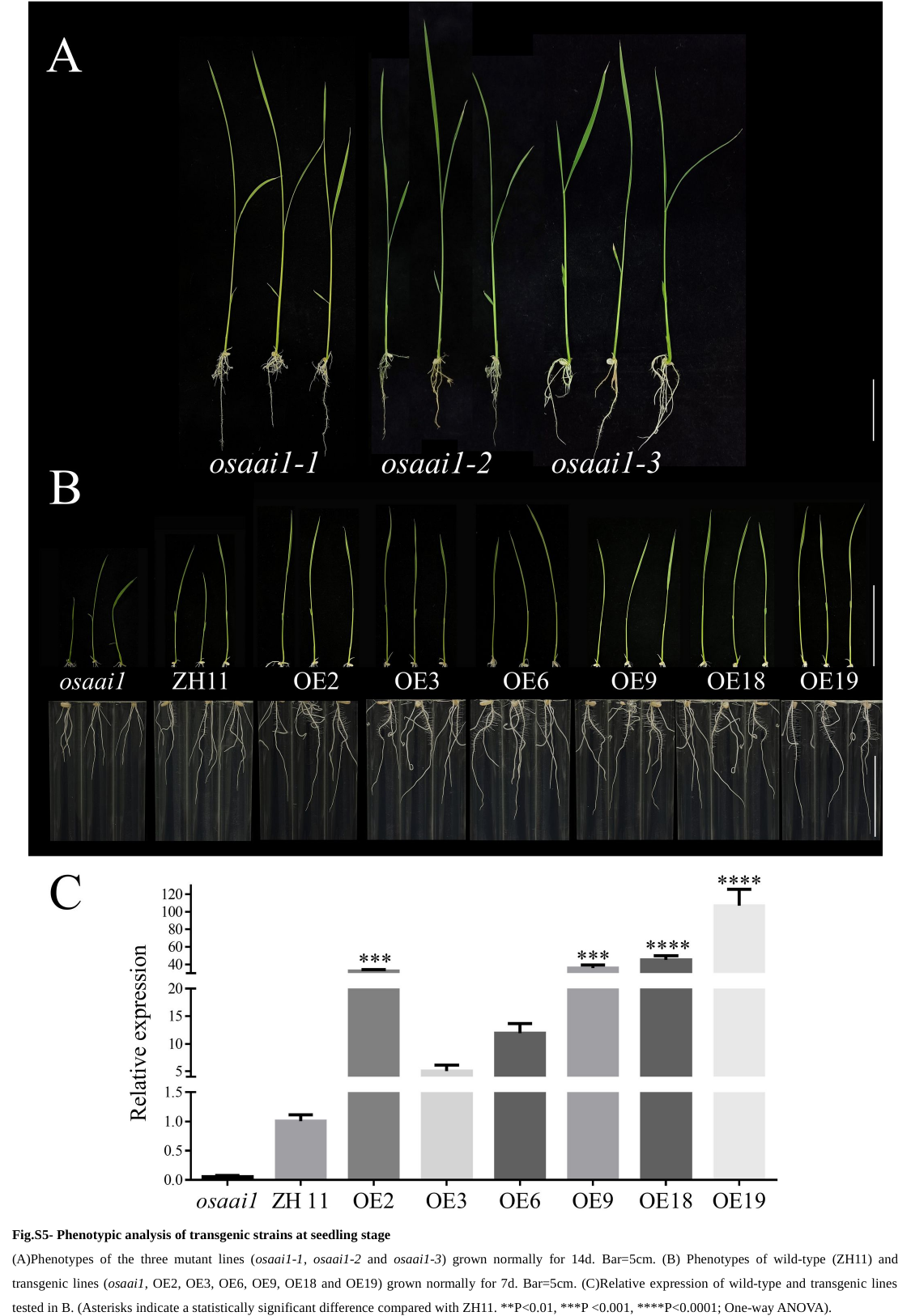

Fig.S5- Phenotypic analysis of transgenic strains at seedling stage
(A)Phenotypes of the three mutant lines (osaai1-1, osaai1-2 and osaai1-3) grown normally for 14d. Bar=5cm. (B) Phenotypes of wild-type (ZH11) and transgenic lines (osaai1, OE2, OE3, OE6, OE9, OE18 and OE19) grown normally for 7d. Bar=5cm. (C)Relative expression of wild-type and transgenic lines tested in B. (Asterisks indicate a statistically significant difference compared with ZH11. **P<0.01, ***P <0.001, ****P<0.0001; One-way ANOVA).

## Slide 6
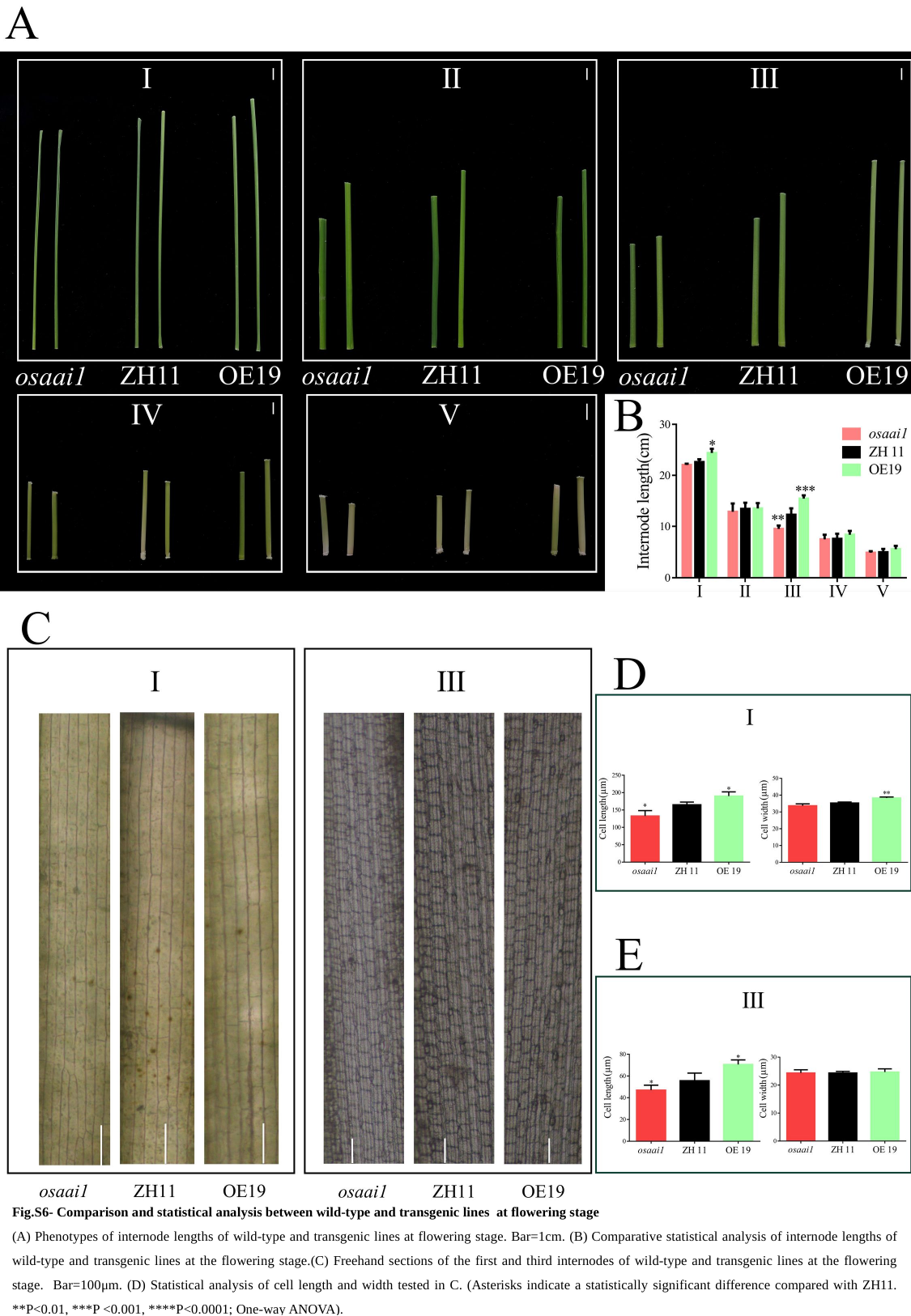

Fig.S6- Comparison and statistical analysis between wild-type and transgenic lines at flowering stage
(A) Phenotypes of internode lengths of wild-type and transgenic lines at flowering stage. Bar=1cm. (B) Comparative statistical analysis of internode lengths of wild-type and transgenic lines at the flowering stage.(C) Freehand sections of the first and third internodes of wild-type and transgenic lines at the flowering stage. Bar=100μm. (D) Statistical analysis of cell length and width tested in C. (Asterisks indicate a statistically significant difference compared with ZH11. **P<0.01, ***P <0.001, ****P<0.0001; One-way ANOVA).

## Slide 7
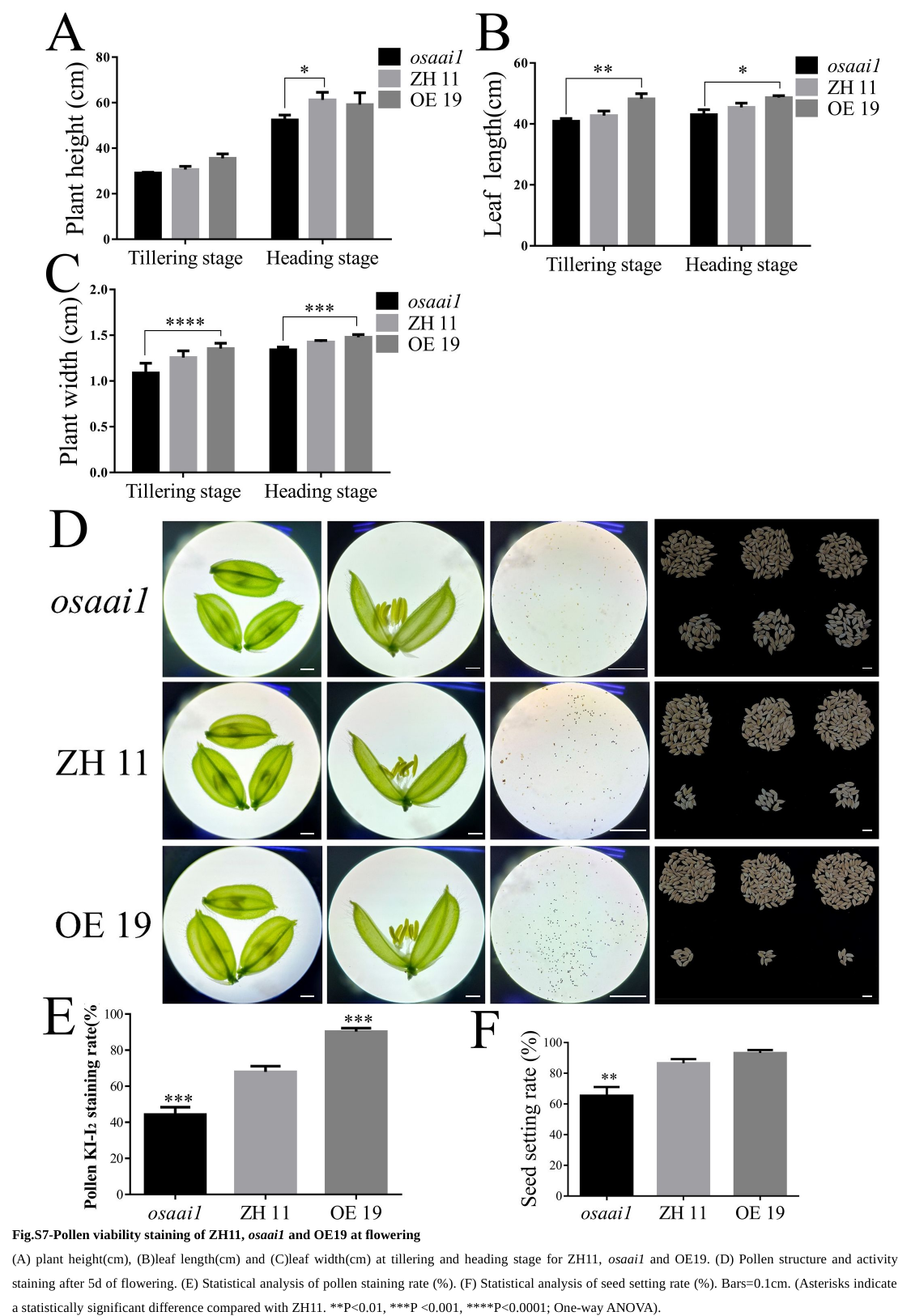

Fig.S7-Pollen viability staining of ZH11, osaai1 and OE19 at flowering
(A) plant height(cm), (B)leaf length(cm) and (C)leaf width(cm) at tillering and heading stage for ZH11, osaai1 and OE19. (D) Pollen structure and activity staining after 5d of flowering. (E) Statistical analysis of pollen staining rate (%). (F) Statistical analysis of seed setting rate (%). Bars=0.1cm. (Asterisks indicate a statistically significant difference compared with ZH11. **P<0.01, ***P <0.001, ****P<0.0001; One-way ANOVA).

## Slide 8
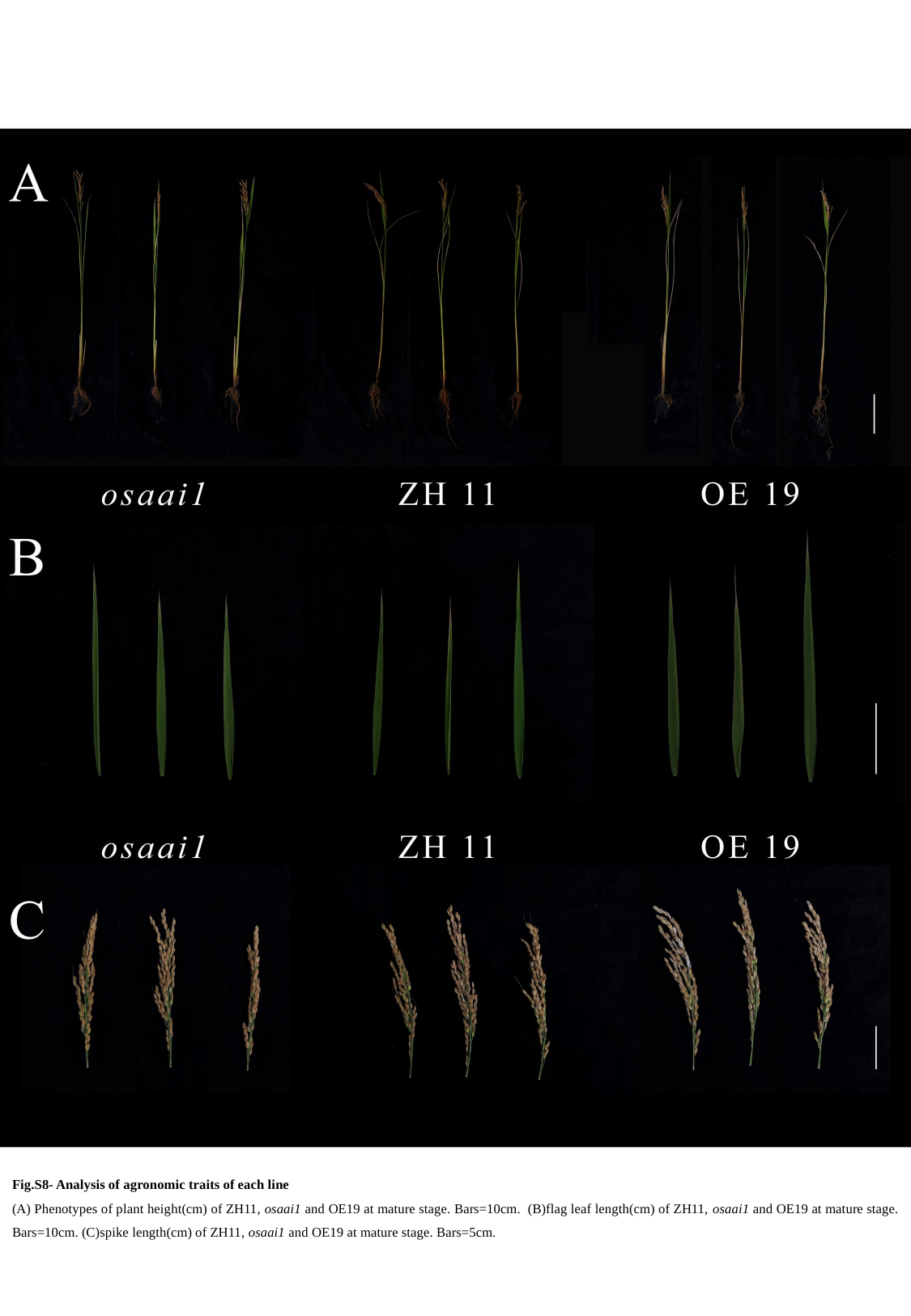

Fig.S8- Analysis of agronomic traits of each line
(A) Phenotypes of plant height(cm) of ZH11, osaai1 and OE19 at mature stage. Bars=10cm. (B)flag leaf length(cm) of ZH11, osaai1 and OE19 at mature stage. Bars=10cm. (C)spike length(cm) of ZH11, osaai1 and OE19 at mature stage. Bars=5cm.

## Slide 9
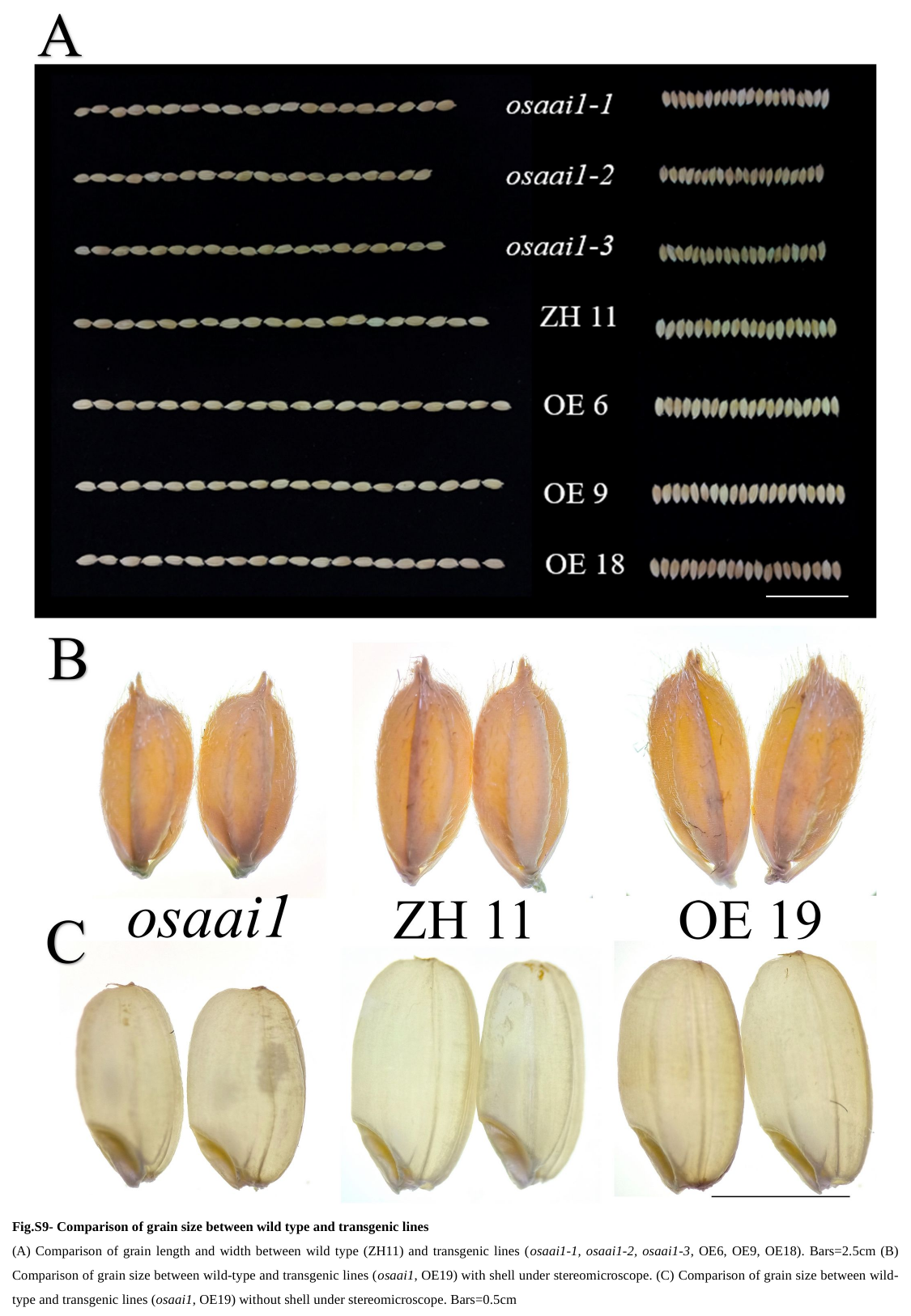

Fig.S9- Comparison of grain size between wild type and transgenic lines
(A) Comparison of grain length and width between wild type (ZH11) and transgenic lines (osaai1-1, osaai1-2, osaai1-3, OE6, OE9, OE18). Bars=2.5cm (B) Comparison of grain size between wild-type and transgenic lines (osaai1, OE19) with shell under stereomicroscope. (C) Comparison of grain size between wild-type and transgenic lines (osaai1, OE19) without shell under stereomicroscope. Bars=0.5cm

## Slide 10
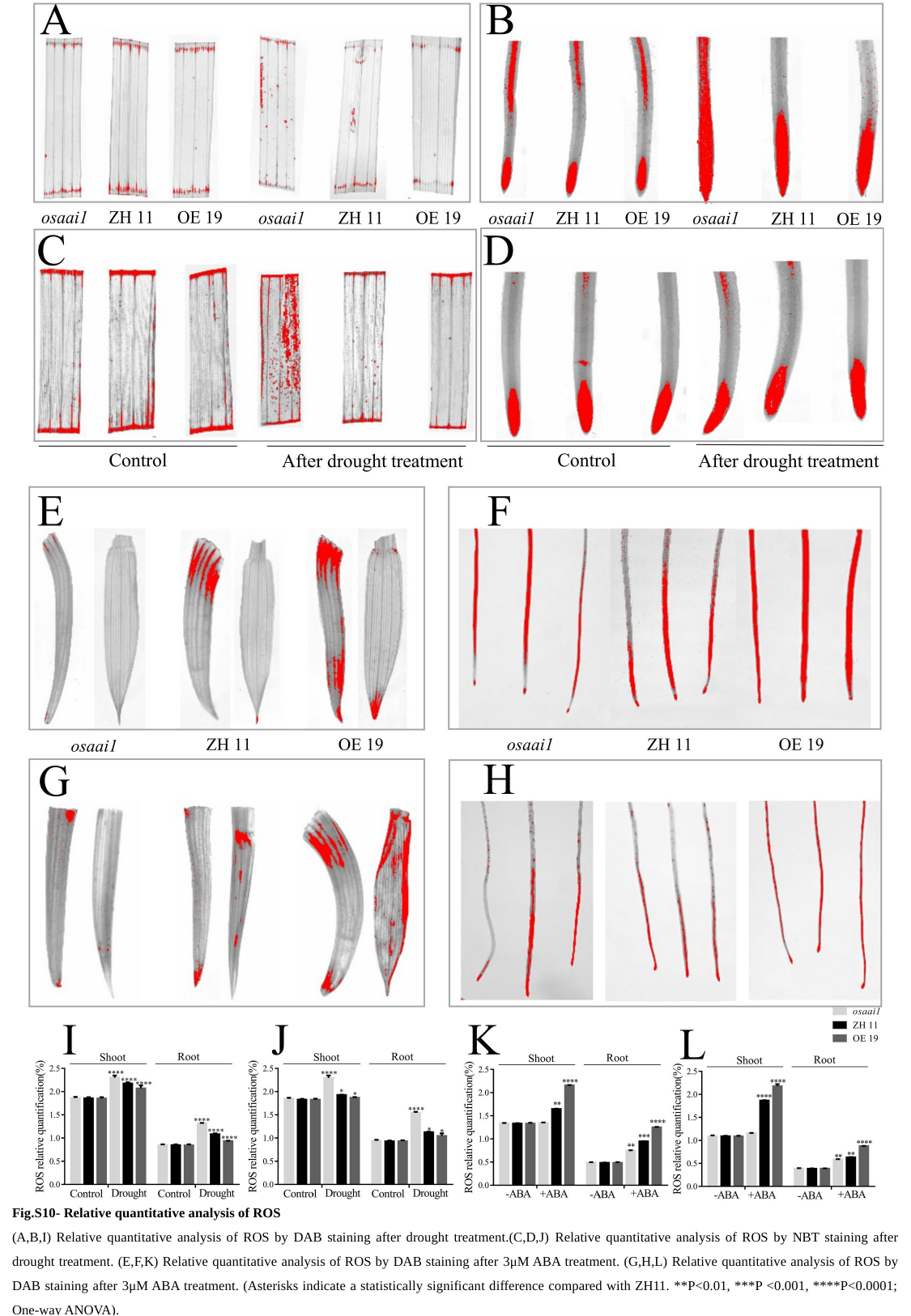

Fig.S10- Relative quantitative analysis of ROS
(A,B,I) Relative quantitative analysis of ROS by DAB staining after drought treatment.(C,D,J) Relative quantitative analysis of ROS by NBT staining after drought treatment. (E,F,K) Relative quantitative analysis of ROS by DAB staining after 3μM ABA treatment. (G,H,L) Relative quantitative analysis of ROS by DAB staining after 3μM ABA treatment. (Asterisks indicate a statistically significant difference compared with ZH11. **P<0.01, ***P <0.001, ****P<0.0001; One-way ANOVA).
